# Microbial community development during syngas methanation in a trickle bed reactor with various nutrient sources

**DOI:** 10.1007/s00253-022-12035-5

**Published:** 2022-07-08

**Authors:** George Cheng, Florian Gabler, Leticia Pizzul, Henrik Olsson, Åke Nordberg, Anna Schnürer

**Affiliations:** 1 Department of Molecular Science, Biocenter SLU, Box 7015, 750 07 Uppsala, Sweden; 2 Department of Energy and Technology, SLU, Box 7032, 750 07 Uppsala, Sweden; 3 Department of Biorefinery and Energy, RISE, Box 7033, 750 07 Uppsala, Sweden

**Keywords:** Methanation, Syngas, Microbial community, *Sporomusa*, *Methanobacterium*, Trickling bed reactor

## Abstract

**Abstract:**

Microbial community development within an anaerobic trickle bed reactor (TBR) during methanation of syngas (56% H_2_, 30% CO, 14% CO_2_) was investigated using three different nutrient media: defined nutrient medium (241 days), diluted digestate from a thermophilic co-digestion plant operating with food waste (200 days) and reject water from dewatered digested sewage sludge at a wastewater treatment plant (220 days). Different TBR operating periods showed slightly different performance that was not clearly linked to the nutrient medium, as all proved suitable for the methanation process. During operation, maximum syngas load was 5.33 L per L packed bed volume (pbv) & day and methane (CH_4_) production was 1.26 L CH_4_/L_pbv_/d. Microbial community analysis with Illumina Miseq targeting 16S rDNA revealed high relative abundance (20–40%) of several potential syngas and acetate consumers within the genera *Sporomusa*, *Spirochaetaceae*, *Rikenellaceae* and *Acetobacterium* during the process. These were the dominant taxa except in a period with high flow rate of digestate from the food waste plant. The dominant methanogen in all periods was a member of the genus *Methanobacterium*, while *Methanosarcina* was also observed in the carrier community. As in reactor effluent, the dominant bacterial genus in the carrier was *Sporomusa*. These results show that syngas methanation in TBR can proceed well with different nutrient sources, including undefined medium of different origins. Moreover, the dominant syngas community remained the same over time even when non-sterilised digestates were used as nutrient medium.

**Key points:**

**•** *Independent of nutrient source, syngas methanation above 1 L/L*_pbv_*/D was achieved.*

**•** *Methanobacterium and Sporomusa were dominant genera throughout the process.*

**•** *Acetate conversion proceeded *via* both methanogenesis and syntrophic acetate oxidation.*

**Graphical abstract:**

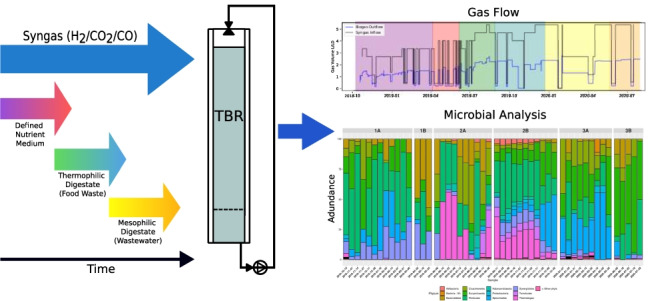

**Supplementary Information:**

The online version contains supplementary material available at 10.1007/s00253-022-12035-5.

## Introduction

Anaerobic digestion is a well-established and well-known process-based technology for treatment of different types of organic waste streams, such as sewage sludge, manure or food waste, while producing renewable energy (biogas) and a nutrient-rich digestate that can be used as fertiliser (Kougias and Angelidaki 2018). Among possible substrates for biogas production, plant biomass residues, such as straw, represent a huge global resource with great potential (Paul and Dutta 2018). However, such materials are currently rather under-used in biogas reactors due to limited applicability within the conventional digestion process (Hendriks and Zeeman 2009; Paul and Dutta 2018). One way to tap the potential of such materials can be thermal gasification to syngas, followed by conversion to methane (Ren et al. 2020).

The composition of syngas varies depending on biomass type and gasification conditions, but it mainly consists of methane (CH_4_), carbon dioxide (CO_2_), hydrogen (H_2_) and carbon monoxide (CO), with very low concentrations of nitrogen (N_2_) and oxygen (O_2_) and trace gases in varying amounts (Ciliberti et al. 2020). Although syngas can be combusted directly, conversion to an established biofuel, such as methane, offers synergies with existing infrastructure for energy storage and distribution (Ren et al. 2020). Methanation of syngas can be performed by chemical catalytic methods or biologically (biomethanation) using methanogenic archaea (Ren et al. 2020). Biomethanation has the advantage that it can be carried out at ambient operating conditions (low pressure, low temperature) (Asimakopoulos et al. 2020b; Aryal et al. 2021; Wegener Kofoed et al. 2021). Moreover, compared with the chemical conversion process biomethanation is more robust to impurities, such as tar or hydrogen sulphide (H_2_S) (Grimalt-Alemany et al. 2018). Based on this, biomethanation is estimated to be more cost-efficient than chemical catalytic conversion (Benjaminsson et al. 2013).

One challenge for the biomethanation process is gas–liquid transfer. Among different reactor systems available, the trickle bed reactor (TBR) can be considered an efficient technology for producing biomethane from syngas (Sposob et al. 2021). The TBR consists of a column packed with carrier material with high surface area, for immobilisation of microbial biomass. A liquid nutrient medium to support microbial growth and activity is sprinkled at the top of the TBR and trickles over the carrier material to the bottom of the reactor, while input gas flows in a counter-current or co-current direction. Rate-limiting mass transfer of gases is circumvented in TBR, since the carrier material provides a large surface area for interactions between gas, liquid and biofilm (Sposob et al. 2021). However, combining TBR and biomethanation is a relatively new concept and parameters indicating a stable continuous process and high output have not yet been identified (Grimalt-Alemany et al. 2017).

**Fig. 1:**
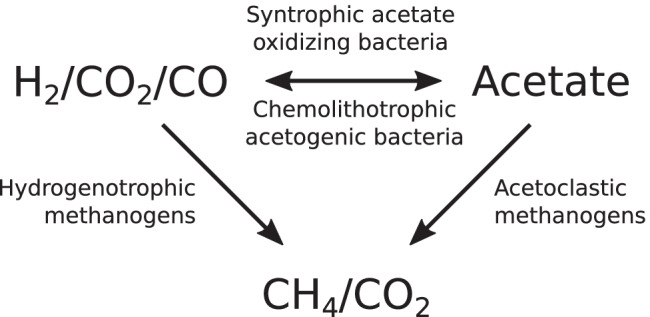
Groups of microbes involved in methanation of syngas. Hydrogenotrophic methanogens converting H_2_/CO_2_ to methane (CH_4_) also compete with chemolithotrophic acetogenic bacteria that consume H_2_/CO_2_ to produce acetate. Acetate is consumed by either acetoclastic methanogens for CH_4_ production or syntrophic acetate oxidising bacteria to produce H_2_/CO_2_

Acetogenesis and methanogenesis are two main essential microbial routes during methanation of syngas or H_2_/CO_2_ (Grimalt-Alemany et al. 2018), although syntrophic acetate oxidation (SAO) can also be part of the process (Sancho Navarro et al. 2016) (Fig. 1). Hydrogenotrophic methanogens convert H_2_ and CO_2_ to CH_4_, but the same substrates can be used by acetogens for the production of acetate, creating competition for H_2_. In addition, CO can be used by acetogenic bacteria (Arantes et al. 2020) but can also be converted by some methanogens (Ferry 2010; Sancho Navarro et al. 2016). Based on thermodynamics and substrate affinities, methanogens have an advantage over acetogens as they can use lower levels of dissolved hydrogen (reviewed in Wegener Kofoed et al. 2021). However, acetogens become more competitive at lower operating temperatures (Fu et al. 2019), higher hydrogen levels (Liu et al. 2016) and high or low pH values, which inhibit methanogens (Voelklein et al. 2019). Acetate can be consumed by acetoclastic methanogens to produce methane or oxidised to H_2_/CO_2_ by syntrophic acetate-oxidising bacteria. The latter process is preferentially operating under low partial pressures of hydrogen and carbon dioxide and thus are likely to have a negligible role during methanation of syngas. However, under certain conditions syntrophic acetate oxidation can be enabled and compete with acetoclastic methanogenesis, such as during low P_CO2_ levels (< 0.01 atm), which improves the thermodynamics of this metabolic route (Grimalt-Alemany et al. 2020a), and high P_CO_ concentrations, which inhibits acetate utilising methanogens (Sancho Navarro et al. 2016).

To ensure high microbiological activity and growth during conversion of gases, such as syngas or H_2_/CO_2_, it is important to supply sufficient amounts of nutrients with the liquid medium (Wegener Kofoed et al. 2021). It is well known that, in addition to carbon and energy sources represented by the process gases, microorganisms (including methane producers) also need other macronutrients and micronutrients, such as nitrogen (N), phosphorus (P), sulphur (S) and various salts and trace metals (Jarrell and Kalmokoff 1988). Several previous studies have examined syngas methanation in laboratory-scale TBR operating with different defined nutrient media (Burkhardt et al. 2015; Asimakopoulos et al. 2020a, b). For full-scale application, there is a need for more accessible and economically feasible nutrient sources, such as manure, digestate or reject water from sludge processing in wastewater treatment plants (WWTP). The use of such undefined nutrient media has mainly been evaluated for biomethanation of H_2_/CO_2_, during operation in batch or continuous mode (Kougias and Angelidaki 2018; Sieborg et al. 2020; Tsapekos et al. 2021), and for biomethanation of syngas in a TBR in batch mode (Aryal et al. 2021). To our knowledge, only one previous study has used a non-defined nutrient source during continuous operation of a TBR for syngas methanation (Figueras et al. 2021).

The biological processes involved in syngas conversion have been investigated in many studies and have been shown to be influenced by different parameters, such as type of reactor and carrier, composition of the gas, environmental parameters such as temperature, and liquid recirculating rates and nutrient composition (for reviews, see Grimalt-Alemany et al. 2017; Aryal et al. 2021; Li et al. 2021; Sposob et al. 2021; Tsapekos et al. 2021). Other determinants of process efficiency, such as microbial community structure and abundance as influenced by operating parameters, have been less thoroughly investigated and the links between operational settings and performance and the microbial community are not fully understood. The few microbiological analyses that have been performed have mainly focused on methanation from H_2_/CO_2_ (Sposob et al. 2021; Tsapekos et al. 2021) and less on syngas, and most studies have been performed at thermophilic temperatures, using defined medium (Li et al. 2020a, 2021; Andreides et al. 2021; Aryal et al. 2021). The aim of the present study was to extend knowledge on the microbiology of syngas methanation and, more specifically, to investigate the influence of different nutrient media on microbial community development during long-term operation of a mesophilic TBR. The work formed part of a larger research project and was performed over 3 years in a succession of periods utilising defined and complex nutrient medium, represented by digestate from a food waste–based biogas plant and reject waster from a WWTP. The syngas used in the study was designed to mimic the expected composition of syngas according to an industrial partner, Cortus Energy Ltd.

## Material and methods

### Screening and selection of inoculum

Initial screening of syngas consumption capacity, using digestate from three different mesophilic biogas reactors (a, b, c), was performed to select a suitable microbial inoculum for the start-up of the TBR. Reactors a and b were mesophilic laboratory-scale reactors, operating with cow manure and mixed food waste, respectively. Operation and performance of these reactors have been described elsewhere, as manure-based Reactor A_ref_ in Ahlberg-Eliasson et al. (2021) and food waste reactor GR2 in Westerholm et al. (2015), respectively. The third inoculum was taken from a full-scale reactor (c), located at Uppsala WWTP, operating with a mix of secondary and primary sludge at mesophilic temperature (37 °C), using an organic load of 2 g volatile solids/L day and a hydraulic retention time of 18 days. The volatile solid concentration (% of wet weight) in inoculum from reactor a, b and c was 6.1, 2.9 and 1.5%, respectively. Each inoculum was incubated at 37 °C for 7 days to remove excess gas from endogenous material and then 200 mL of inoculum was transferred to each of 12 serum bottles (539.5 mL) under flushing with nitrogen gas. The bottles were sealed with butyl rubber stoppers and aluminium caps and filled with different gases to one of the following concentrations (%) at a final pressure of 1.5 atm: (i) CO/N_2_ (15/85%); (ii) CO/H_2_/CO_2_/N_2_ (15/28/7/50%); (iii) H_2_/CO_2_ (28/72%); (iv) N_2_ (100%), with three replicates per gas composition. The gases used were synthetic mixtures supplied by Air Liquide (Paris, France). The bottles were then incubated at 37 °C for 10 days on a shaking table at 200 rpm (Orbitron, Infors, Bottmingen, Switzerland). Gas compositional analyses were performed after 1, 2, 3, 7, 8 and 10 days. On each sampling occasion, the pressure was measured (model GMH3111; Greisinger Electronics, Regenstauf, Germany) and a 5 mL gas sample (at normal pressure) was taken with a plastic syringe. The gas was analysed by gas chromatography (see below). Based on the initial screening of syngas consumption (Fig. S1), inoculum from the manure-based reactor (reactor a) was selected as the inoculum source.

Digestate collected from reactor a (inoculum A) was filtered through a 2-mm mesh to remove large particles, after which 1.25 L was diluted with defined mineral medium (Westerholm et al. 2010) to reach a final volume of 5 L. The diluted digestate was transferred under flushing (N_2_) to two plastic containers (20 L), each filled with 17.5 L plastic carrier (Hiflow® ring 15–7 plastic; height 15 mm, specific area 313 m^2^/m^3^, density 80 kg/m^3^, void fraction 91%). The containers were closed and incubated anaerobically at 37 °C for 7 days, with manual shaking twice every day. This incubation was intended to reduce the level of organic matter in the inoculum, decrease background CH_4_ production and initiate biofilm development on the carrier before filling the TBR.

### Source of nutrients

During operation of the TBR, three different nutrient sources were used: defined medium (M1); digestate from a thermophilic biogas plant (Uppsala, Sweden) operating with mixed food waste (Grim et al. 2015) (M2); and reject water from dewatered digestate from a biogas unit at a WWTP (Höganäs, Sweden) operating with mixed primary and activated sludge (M3). Medium M1 was prepared as described previously (Westerholm et al. 2010) and consisted of phosphate/bicarbonate buffer supplemented with salt, trace metals, vitamins and reducing agents (e.g. cysteine-hydrochloric acid (HCl) and sodium sulphide (NaS_2_)). These reducing agents also represented the only S source in the medium, with a total S concentration of 135 mg/L. The N source in the medium was ammonium chloride (NH_4_Cl), with an ammonium-N concentration of 400 mg/L. The pH of M1 was 7.2–7.4. Medium M2 was prepared by mixing one volume unit of digestate with two volume units of deionised water. The resulting liquid was passed through a cloth to remove particles. No sterilisation of the liquid was performed. This diluted solution had a pH of 8.5–8.6, total alkalinity of 2249–3057 mg/L and an ammonium-N concentration of 300–670 mg/L. Sulphur was analysed as sulphate and the concentration was 50–60 mg/L. The reject water medium (M3) was dewatered but not sterilised at the WWTP and no further treatment was done before its use as nutrient medium for the TBR. The pH was 7.6–7.9, the total alkalinity was 2676–3871 mg CaCO_3_/L and the concentration of ammonium-N and S was 560–800 mg/L and 107 mg/L, respectively. Additional supplements were added to the media as described in section TBR Operation

### TBR and anaerobic filter

**Fig. 2:**
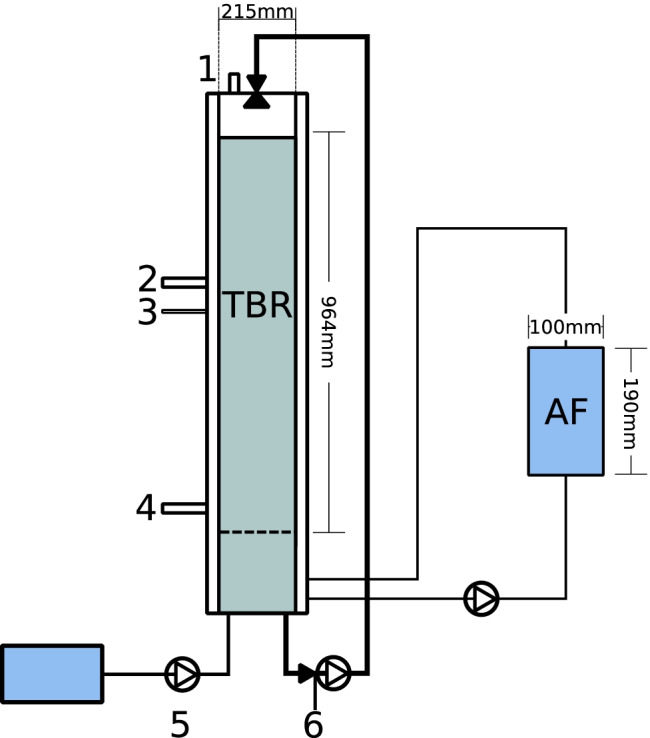
Schematic diagram of the trickle bed reactor (TBR) and anaerobic filter (AF). 1: Outlet for product gas. 2: Carrier sampling port. 3: Position of temperature probe. 4: Syngas inflow. 5: Inflow of liquid nutrient medium. 6: Sampling port for microbial analysis

The TBR was constructed from acid-proof stainless steel (Fig. 2) and was placed in a movable container together with all associated equipment (Fig. S2). The reactor had a total volume of 49 L, including head space and liquid reservoir, an inner diameter of 215 mm and a total height of 1344 mm. The reactor was filled with the inoculated carrier to a total packed bed volume (pbv) of 35 L. A grid plate spreader (5 mm pores) was placed 80 mm above the bottom of the reactor, creating a reservoir in which a volume of nutrient medium (max. 8 L) could be contained and collected. Liquid from this reservoir was manually removed on a regular basis and replaced with fresh nutrient medium. The liquid was recirculated with a hose pump (FPSH 15, 0.37 kW; Valisi, Rozzano, Italy) from the reservoir to the top of the reactor, where it was sprinkled over the packed bed and trickled back to the liquid reservoir. The pump was operated in semi-continuous batch mode with 5 s of pumping followed by 37 s of stop time, giving an average flow of approximately 14.3 L/h. At the top of the reactor, two stainless steel grid plate spreaders were positioned to distribute the nutrient liquid (Fig. 2, Fig. S2d). Syngas was added through a port in the lower part of the reactor (Fig. 2) to meet the liquid coming from the top, thus operating in a counter-current manner. At the top of the reactor, the gas was collected and the volume was measured by a drum meter (TG 0.5; Ritter, Germany). Samples for chemical and microbiological analyses of the liquid were taken at position 6 in Fig. 2. The composition of the outgoing gas (CH_4_, CO_2_, CO, O_2_, H_2_) was analysed by a ETG MCA 100 Syn Biogas Multigas Analyzer (ETG Risorse e Tecnologia, Chivasso, Italy). The reactor was heated to 37 °C by a water jacket and the temperature in the reactor was logged using a temperature probe (Tinytag View 2; Gemini Data loggers, Chichester, United Kingdom). The temperature was 36–38 °C during the entire operating period of the TBR. After around 200 days of operation, an additional reactor (anaerobic filter, AF) was installed, through which nutrient liquid from the bottom of the TBR was recycled in an upflow manner (Fig. 2, Fig. S2d). The aim was for the AF to prolong the retention time for the nutrient liquid recirculate and by doing so allow more time for degradation of accumulated volatile fatty acids (VFA) in the nutrient liquid. This reactor was made of plastic and had a total/active volume of 1.5 L (height 190 mm, diameter 100 mm). The AF was filled with the same type of inoculum and carrier as the TBR and the same procedure as for TBR inoculation was used, with an incubation period of 14 days before filling the AF. The gas from this reactor was not collected.

### TBR operation

The TBR was operated for a total of 862 days. The initial 129 days of operation were devoted to start-up and acclimatisation of the process. Thereafter, TBR operation was divided into different main periods based on the nutrient medium used (Table 1). Each main operating period was in turn divided in two sub-phases (A and B) based on major operational changes, installation of the AF or changes in flow or composition of the nutrient medium (Table 1). The gases used throughout TBR operation were synthetic mixtures (Air Liquide, Paris, France). The different periods are described briefly below and summarised in Table 1 and Supplementary Material.

**Table 1:** Description of the different operating periods in the trickle bed reactor (TBR) process

Phase name	Period dates	Days of operation^a^	Nutrient solution^b^	Feed rate^c^ (mL/day)	Gas composition^d^ (%)	Description
Start-up	2018/06/01–2018/07/26	− 129 (56)	M1^e^	140	CO: 15 N_2_: 85	Initiation of reactor and enrichment of CO-utilising microorganisms
Acclimatisation	2018/07/27–2018/10/07	− 73 (73)	M1^e^	140	CO: 15 CO_2_: 7 H_2_: 28 N_2_: 50	Change of gas mixture towards industrial-like gas composition and acclimatisation
Period 1A	2018/10/08–2019/04/04	0–179 (179)	M1^e^	140	CO: 30 CO_2_: 14 H_2_: 56	Change of gas mixture to simulate syngas produced by Cortus Energy. This gas mixture was used in the following periods
Period 1B	2019/04/05–2019/06/05	180–241 (62)	M1^e^	140	CO: 30 CO_2_: 14 H_2_: 56	Addition of a small anaerobic filter (AF) reactor to alleviate accumulating VFA levels. The small reactor was used to the end of the process
Period 2A	2019/06/06–2019/08/27	242–324 (83)	M2^f^	1000	CO: 30 CO_2_: 14 H_2_: 56	Change in liquid nutrient feed stabilisation and feed rate
Period 2B	2019/08/28–2019/12/22	325–441 (117)	M2^f^	1000→200	CO: 30 CO_2_: 14 H_2_: 56	Gradually decrease in liquid feeding throughout phase
Period 3A	2019–12-23– 2020/05/19	442–590 (149)	M3^g^	400	CO: 30 CO_2_: 14 H_2_: 56	Change in liquid nutrient feed and feed rate
Period 3B	2020/05/20–2020/07/29	591–661 (71)	M3^g^	200	CO: 30 CO_2_: 14 H_2_: 56	Nutrient feed rate reduced to 200 mL

^a^Operating time set to zero at the start of period 1; days in brackets represent the number of operation days for each period

^b^Recirculated liquid nutrient solution

^c^Feeding rate of liquid nutrient medium

^d^Composition of ingoing gas mixture

^e^Defined nutrient medium

^f^Digestate from a co-digestion plant, operated under thermophilic conditions, digesting sorted household food waste with organic food waste from larger kitchens, stores and food distributors

^g^Digestate from a wastewater treatment plant, operated under mesophilic conditions, digesting sludge from the wastewater treatment process and minor fractions of different sludges from the food processing industry

#### Start-up and acclimatisation (129 days)

During this period, the biomass was allowed to adjust to the prevailing conditions in the reactor and to the syngas. Defined nutrient medium (M1) was used at a low flow rate (140 mL/day) and the inlet gas composition was initially CO/N_2_ (15/85), to enrich CO-consuming bacteria. Thereafter, the gas composition was changed to CO/CO_2_/H_2_/N_2_, representing 15, 7, 28 and 50%, respectively, in order to add CO_2_ and H_2_ while maintaining the same partial pressure of CO.

#### Period 1 (241 days)

The start of this operating period was defined as Day 0. In this period, the reactor was fed syngas with the target composition expected by the industrial partner (Cortus Energy), which was 30% CO, 14% CO_2_ and 56% H_2_. The defined nutrient medium (M1) at an average inflow rate of 140 mL/d was used throughout. During operation, VFA were produced quickly in response to increasing syngas inflow, so an AF was installed (phase 1B) and process liquid from the TBR reservoir was recirculated through the TBR (Fig. 1). To evaluate possible nutrient limitation as a cause of VFA accumulation and decreasing gas consumption, additional N (NH_4_Cl; Fisher Chemicals, Göteborg, Sweden) and S (NaS_2_ and cysteine-HCl; Merck, Darmstadt, Germany) were also added at the end of period 1A and during period 1B (see Supplementary Material).

#### Period 2 (200 days)

In this period, the nutrient medium was changed to digestate from the industrial food waste biogas plant (M2). In the initial phase (2A), a high flow of nutrients was supplied (1000 mL/day), while in the second phase (2B), this was gradually reduced to 200 mL/day. In phase 2B, additional S (NaS_2_ or NaSO_4_, Merck, Darmstadt, Germany) was added (Table 1, Supplementary Material) in an attempt to mitigate decreasing syngas consumption rate.

#### Period 3 (220 days)

In this period, the nutrient medium was changed to reject water from dewatered digested WWTP sewage sludge (M3)**,** initially at a flow rate of 400 mL/day (phase 3A) and later reduced to 200 mL/day (phase 3B). In addition, extra S (NaS_2_ or NaSO_4_, Merck, Darmstadt, Germany) was added throughout the whole operating period (Supplementary Material) and in phase 3B, sodium bicarbonate (Na_2_CO_3_) was added to enhance the alkalinity and mitigate a trend of decreasing pH (Supplementary Material).

### Analytical methods

In the screening experiments with different inocula, the gas composition was analysed by gas chromatography according to Westerholm et al. (2012). Short-chain VFA (C2-C6) were quantified by ion-exclusion chromatography according to Westerholm et al. (2012). Process pH was measured with a Hanna instrument HI83141 (Woonsocket, Rhode Island, United States). Ammonium and sulphate were analysed with a spectrophotometer (Spectroquant® Nova 60A photometer; MilliporeSigma, Burlington, MA, USA) with reagent test kits from the series Supelco (Merck, Darmstadt, Germany). The total alkalinity was calculated as the amount of acid required to bring the sample to pH 4.4. Titration was carried out with an automatic titrator (TitraLab® AT1000 series; Hach, Düsseldorf, Germany).

### Microbial analysis

Samples for DNA extraction were withdrawn from the recirculated liquid on a weekly basis from sampling port 6 as shown in Fig. 2, and on a few occasions, samples of carrier were taken from the TBR and microbial material was scraped off the carrier using a small spatula. DNA was extracted from 200 μL of liquid sample using the FastDNA Spin Kit for Soil (MPBiomedicals, Illkirch-Graffenstaden, France) according to the manufacturer’s protocol with an additional cleaning step with guanidine thiocyanate (Danielsson et al. 2017). DNA was also extracted from the ingoing nutrient medium by concentrating 4 mL of sample by centrifugation and dissolving the cell pellet obtained in 200 μL of the supernatant. The samples were initially extracted in triplicate but, after preliminary sequence analysis showing no significant variations between triplicate extractions, single extractions were done in order to allow analysis of more samples. Sequencing libraries were generated by SciLifeLab, in Stockholm, Sweden, using Illumina MiSeq (2 × 300 bp) targeting 16S rDNA. To cover both bacteria and archaea, the amplification was done using the forward primer 515′F and reverse primer 806R, as described previously (Westerholm et al. 2018). The paired end reads were processed with Cutadapt version 1.13, removing the aforementioned primers and adapters on forward and reverse reads (GTGBCAGCMGCCGCGGTAA and GACTACHVGGGTATCTAATCC, respectively) and filtering based on quality and trimming reads to 300 bp. The trimmed reads were processed with Division Amplicon Denoising Algorithm2 (DADA2) version 1.16.0 in Rstudio running R version 4.1.1, as described by Westerholm et al. (2018), with forward and reverse reads truncated at positions 240 and 160, respectively. Microbial classification was performed using the SILVA reference database v. 132. The data were organised with *phyloseq* v1.32.0 (McMurdie and Holmes 2013) into a single data object that was subsequently used for graphic generation in RStudio v1.4.1717 (RStudio Team 2020) running R v4.1.1. The following R packages were used for visualisation of the microbial data: ggplot v2 3.3.5, data.table v1.13.4, plotly v4.9.2.1, lattice v0.20.45, permut v0.9.5, vegan v2.5.7, readxl v1.3.1, plyr v1.8.6, grid v4.1.1 and ggtext v0.1.1. The amplicon sequence variants (ASV) were submitted to the Basic Local Alignment Search Tool (BLAST) algorithm provided by the National Center for Biotechnology Information (NCBI), The sequences obtained by Illumina sequencing are too short (~ 250–300 bp) to clearly reveal the identity of the engaged microorganisms on species level. However, ASVs showing 100% identity with a known organism are in the presentation referred to the putative species name. Raw sequence data have been deposited in NCBI PRJNA796200.

## Results

### Selection of inoculum

Evaluation of different sources of inoculum for methane production from different gas mixtures (H_2_/CO_2_, CO/N_2_ or H_2_/CO_2_/CO; see Fig. S1) before the start-up of the TBR process revealed that the consumption/production patterns of the different inocula did not differ significantly. However, CO consumption rate, with or without H_2_ and CO_2_, was highest for the inoculum from the manure-based biogas reactor (reactor a; see Fig. S1), and therefore, inoculum A was chosen for the TBR.

### TBR operation: process and microbiology

#### Period 1

**Fig. 3:**
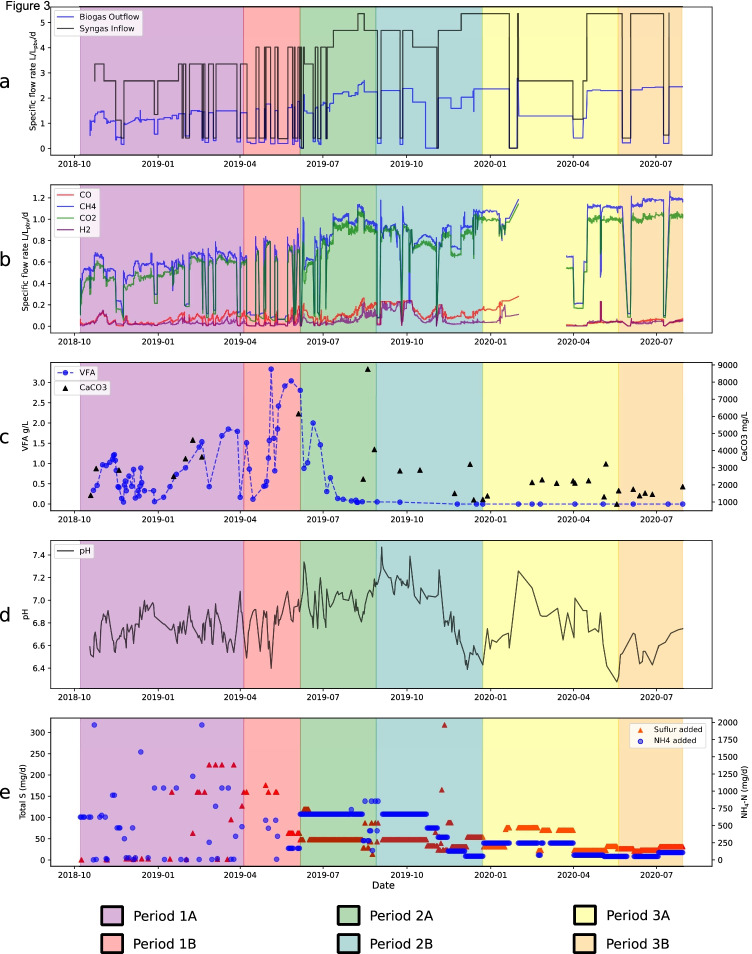
Process data from trickle bed reactor (TBR) operation during three periods (1–3) operating with different nutrient medium: 1) defined medium (M1) 2) dewatered digestate from a thermophilic biogas plant operating with food waste (M2) and 3) reject water from a biogas plant at a wastewater treatment plant (M3). Each period was further divided into two sub phases (A,B) based on major changes in operating parameters, such as flow rate of nutrient medium (see Table 1). **a** Specific syngas inflow (black) and biogas outflow rate (blue). **b** Specific outflow gas rate. The gap seen in period 3A was due to gas analyser malfunction. **c)** Total volatile fatty acids (VFA) concentration and alkalinity. **d** pH. **e** Total amount of sulphur (S) and ammonium nitrogen (NH4-N) added via nutrient medium and by additional supply via external source. For details, see Supplementary Material

Throughout period 1, the syngas inflow fluctuated between 1.11 and 3.33 L/L_pbv_/d, depending on the consumption efficiency (Fig. 3a). During periods of high syngas gas outflow, the inflow was decreased to match the rate of consumption. In line with the variation in inflow, the total biogas outflow for the period ranged from 0.041 to 1.86 L/L_pbv_/d, with higher values towards the end of the period (Fig. 3a). The CH_4_ content in the gas was around 50%, resulting in an output range of 0.06–0.91 L CH_4 _/L_pbv_/d (mean 0.60 L CH_4_/L_pbv_/d) for the period (Fig. 3b). VFA accumulation and pH declines were observed early in operation (Fig. 3c) and the gas inflow was temporarily stopped/lowered on a number of occasions to mitigate further accumulation and decreasing pH. Once VFA were consumed, normal syngas inflow was resumed. However, with an increase in the gas inflow rate, it was no longer possible to control VFA levels, which increased continuously to values as high as 3.7 g/L by the end of period 1B (Fig. 3c, Supplementary Material). The VFA present were mainly represented by acetic and propionic acid, with propionic acid initially making up 71–96%, but with a higher proportion of acetic acid in the later phase (59–99%) (Supplementary Material).

**Fig. 4:**
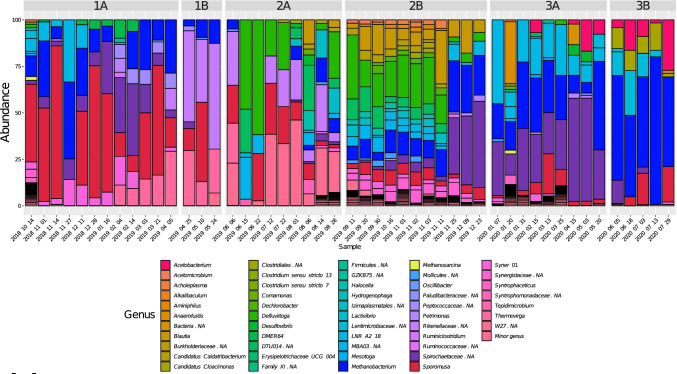
Microbial community structure at genus level during trickle bed reactor (TBR) operation in three periods (1–3) operating with different nutrient medium: (1) defined mineral medium (M1) (2) dewatered digestate from a thermophilic biogas plant operating with food waste (M2) and (3) reject water from a biogas plant at a wasterwater treatment plant (M3). Each operating period was further divided into two sub-phases (A,B) based on major changes in operating parameters, such as flow rate of nutrient medium (see Table 1)

The microbial community in the starting inoculum was characterised by high relative abundance of phylum *Firmicutes* (57.0%), dominated by uncultivated members of genus *MBA03* (15.5%) and genus *Sedimentibacter* (7.7%) and phylum *Bacteriodetes* (24.0%), mainly dominated by unknown members of family *Rikenellaceae* (11.9%) (Fig. 4, Fig. S3a). During the stabilisation phase, these two phyla continued to show high relative abundance, accompanied by emergence of phylum *Syngergistetes* (31.6–50.2%), primarily composed of unknown members of family *Synergistaceae*. The abundance of methanogens (phylum *Euryarchaeota*) was very low in this initial phase of operation and they represented less than < 1% of the total community. However, in period 1, when the H_2_ level in the syngas was increased, the relative abundance of *Euryarchaeota* increased rapidly and for most samples the value was between 10 and 35% (Fig. S3a). Phylum *Euryarchaeota* was represented mainly by one amplicon sequence variant (ASV), which according to a BLAST search in NCBI corresponded to the putative species *Methanobacterium bryantii* (100% similarity). The bacterial community during period 1 also changed compared with that in the stabilisation/acclimatisation phase. Relative abundance of phylum *Bacteriodetes* was initially low but increased when the VFA content was high, represented by two genera within family *Rikenellaceae*, genus DMER64 (1–7%) in period 1A and an unknown genus in period 1B (11–50%). Genus *DMER64* was mainly abundant when propionate represented a major part of the VFA, while the unknown genus was more abundant when propionate level decreased. Phylum *Firmicutes* and phylum *Syngergistetes* showed significant shifts compared with the stabilisation/acclimatisation phase. Throughout period 1, *Firmicutes* was dominated by a member within genus *Sporomusa*, reaching values between 8 and 90% (Fig. 4). A BLAST search of this ASV showed 100% similarity to the putative species *Sporomusa sphaeroides.* Within *Syngergistetes*, genus *Thermovirga* showed an increasing trend over the period. In addition, phylum *Cloacimonetes* and phylum *Spirochaetes* were present during this period, represented mainly by genus *LNR_A2-18* and an unknown member within genus *Spirochaetaceae*, respectively. Genus *LNR_A2-18* initially increased to a high level (~ 23%) in the beginning of period 1A but showed a significant drop just before the start of VFA accumulation (late December 2018) and thereafter remained at low abundance throughout the rest of period 1. *Spirochaetaceae* showed increased relative abundance with a concurrent decrease in relative abundance of *LNR_A2-18*, and represented up to 35% of the community in period 1A, after which this ASV also decreased in abundance. At the time of the decrease, a shift in VFA composition towards a higher level of acetate relative to propionate was seen.

#### Period 2

In period 2, the recirculated nutrient solution was changed from the defined medium (M1) to the digestate from the thermophilic biogas plant operating with mixed food waste (M2) and the nutrient solution feeding rate was increased to 1000 mL/day. The syngas inflow rate in period 2A was initially kept at the same level as in period 1B but, as the VFA level was significantly lower than in period 1, the rate was gradually increased to around 4 L/L_pbv_/d. In line with this increase, the volume of outgoing methane also increased, to reach values of around 0.9–1.10 L CH_4_/L_pbv_/d by the end of period 2A (Fig. 3b). However, towards the end of period 2A, a rise in the outflow levels of CO and H_2_ was observed (Fig. 3b), resulting in a decrease in production of CH_4_. The decreasing trend in CH_4_ production continued during the beginning of period 2B, although VFA were not detected, and the average pH was around 7.2. From the middle to the end of period 2B, the nutrient solution feeding rate was gradually decreased to 200 mL/day, which led to a gradual decrease in NH_4_-N and S supply (Fig. 3c). In an attempt to improve syngas conversion efficiency, which was assumed to be limited by S availability, S was added to the process (Fig. 3e, Supplementary Material). This strategy improved syngas conversion, allowing syngas inflow rate to be increased to 5 L/L_pbv_/d and resulting in an increase in CH_4_ production to around 1.1 L/L_pbv_/d without any accumulation of VFA, although with a decrease in pH to ~ 6.5 (Fig. 3d).

The change of nutrient solution (from M1 to M2) had a major effect on microbial community composition. At the beginning of period 2A, members from the thermophilic phylum *Thermotogae* appeared in high relative abundance, along with *Firmicutes* (Fig. S4a). This community composition mainly reflected the composition of the dewatered digestate used as the nutrient source (Fig. S3a). Phylum *Thermotogae* was represented by genus *Defluviitoga* within the family *Petrotogaceae* (48–56%) and *Firmicutes* were represented by unknown members within two main orders, *DTU014* (6.28–23.49%) and MBA03 (12.36–23.61%)*.* These families stayed in the system until the end of period 2A (Fig. S4b), when the feeding rate of the nutrient medium was reduced to 200 mL/day. In addition, period 2A showed high abundance of phylum *Cloacimonetes* (Fig. S4a), which was not observed in the nutrient solution. This phylum was represented by family *W27* and reached relative abundance values of 17–42% by the end of period 2A (after the decrease in VFA), after which it quickly decreased in period 2B. Moreover, *Sporomusa sphaeroides*, established in period 1 and not present in the digestate, maintained its presence throughout period 2, initially at high relative abundance (~ 20%) but stabilising at lower levels (1.6–6.6%) from the middle of period 2A to the end of period 2B. *Methanobacterium bryantii*, the only methanogen identified in period 2, as in period 1, was present throughout but with increasing relative abundance values towards the end, representing 21–27% of the microbial community (Fig. 4). The community at the end of period 2B was also characterised by high abundance of a member within the genus *Spirochaetaceae* (30–46%), the same species that was dominant in the late stages of period 1.

#### Period 3

In period 3, the nutrient source was switched to reject water from the mesophilic wastewater treatment plant (M3). The feed rate was 400 mL/d during period 3A and decreased to 200 mL/day during period 3B (Table 1). During the whole of period 3, the process was supported with additional S. In addition, sodium hydrogen carbonate (NaHCO_3_) was added to mitigate decreasing pH values, as low as 6.4 during period 3B (Fig. 3e, Supplementary Material). In period 3A, the syngas inflow was initially continued at the same rate as in the previous period, i.e. 5 L/L_pbv_/d, resulting in biogas and methane production of around 2 and 1 L/L_pbv_/d, respectively (Fig. 3a). However, due to problems with the gas analyser, no data were obtained for the outgoing gas for some time and therefore the syngas inflow was decreased to 2.67 L/L_pbv_/d in order to avoid the risk of overloading. When the functionality of the gas analyser was restored, the syngas inflow rate was again set to the previous level, which resulted in similar CH_4_ production values as before. In period 3B, syngas conversion was maintained at a high level, which led to an average CH_4_ production rate of 1.15 L/L_pbv_/d. No VFAs were observed during operation in period 3 (Fig. 3c).

The use of the new medium (M3) in period 3 had little or no influence on the community composition compared with that in period 2. The relative abundance of *Methanobacterium bryantii*, the dominant methanogen, increased in period 3 compared with period 2 and reached values of 21–45% for most of the samples analysed (Fig. 4). For bacteria, the relative abundance of the previously observed member within the genus *Spirochaetaceae* wa*s* initially maintained at a similar level as observed in period 2B, but it decreased gradually when the nutrient flow rate decreased in period 3B, to reach values of 2–3% at the end (Fig. 4). *Sporomusa sphaeroides* was initially high in period 3 but decreased during the pH decrease and then recovered towards the end of period 3B, reaching values of around 10%. In addition, at the end of period 3B, the relative abundance of a member within genus *Acetobacterium* increased and it became one of the most abundant species at the end, representing ~ 31% (Fig. 4). A BLAST search in NCBI showed 100% similarity with the putative species *Acetobacterium wieringae*. In addition, genus LNR_A2-18 (phylum *Cloacimonetes*) was present in high relative abundance (5–26%) throughout period 3. This ASV was the same as that previously identified in high relative abundance in period 1. Another unknown member within this phylum was also identified in period 3B and showed increasing abundance towards the end, representing 12% of the total community. This increase in abundance was the opposite of the decreasing trend seen for *Sporomusa sphaeroides* during the stage of decreasing pH.

### Microbial analysis of carrier samples

**Fig. 5:**
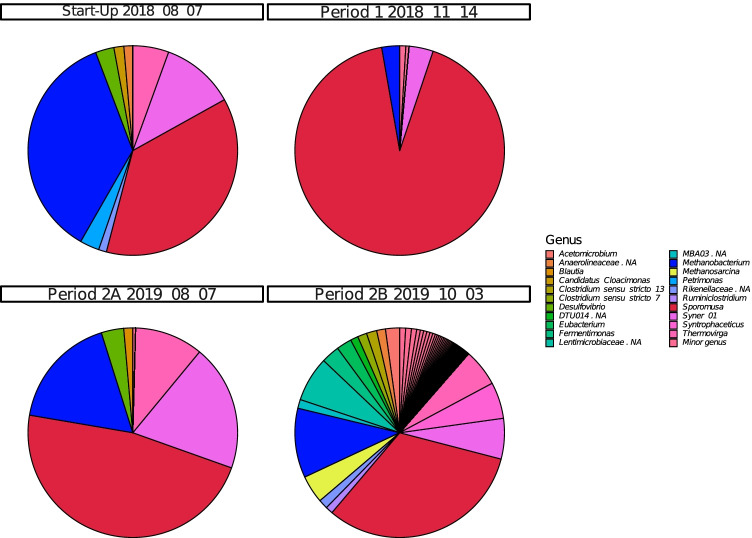
Microbial community structure on genus level in biofilm recovered from plastic carriers in the trickle bed reactor (TBR) in the start-up phase and in operating periods 1 and 2. Carrier samples taken in period 2B were sequenced in triplicate. Carrier samples taken in start-up, period 1 and period 2A were sequenced without replicates, due to lack of extracted material

Carrier samples were taken on only four occasions and only from one position (Fig. 2), due to the difficulty in removing the carrier without causing process disturbance. Independent of sampling time, *Firmicutes* was one of the most highly abundant phyla on the carrier samples, with relative abundance ranging from 37 to 92%, primarily represented by *Sporomusa* at the genus level (Fig. 5). *Euryarchaeota* was present in all carrier samples (Fig. S5), represented mainly by *Methanobacterium* (3–36%), but at the final carrier sampling point in the process (phase 2B), *Methanosarcina* (4%) was also detected (Fig. 5). Phylum *Synergistetes* (4–30%) maintained a presence on the carrier through the four sampling points (Fig. S5) and was comprised mainly of genus *Syner-01* (3–19%) and *Thermovirga* (5–10%) at the first three sampling points. At the final sampling point in period 2B, an unknown genus in *Syngeristaceae* (0.21%) was observed together with *Acetomicrobium* (2.2%) and *Aminobacterium* (0.49%) (Fig. S5). At the final sampling point, there was also comparatively high relative abundance of Bacteroidetes (15%), represented by genus *Fermentimonas* (3%, family *Dysgonomonadaceae*) and a genus belonging to family *Lentimicrobiaceae* (7%) (Fig. 5, Fig. S5b). The ASV belonging to the *Sporomusa* found on all carrier samples was identified as *Sporomusa sphaeroides*, with 99.2% similarity in a BLAST search. The ASV for *Methanosarcina* showed 99.2% similarity with *Methanosarcina flavescens.*

## Discussion

### Selection of start-up inoculum

The inoculum for the TBR was chosen from an initial screening of three different digestates. The selection of digestate was based on the processes having different microbial communities and dominance of different methane-producing pathways, potentially influencing syngas consumption capacity. It is known that microbial community structure in processes operating with sludge, manure and food waste is different and statistically distinct, mostly driven by differences in ammonia levels (Sundberg et al. 2013; De Vrieze et al. 2015). The food waste reactor (reactor b), operating at a high ammonia level (0.5–0.9 g NH_3_/L), was shown to have high abundance of the hydrogentrophic methanogen *Methanoculleus bourgensis* (genus *Methanomicrobiales*) and different known syntrophic acetate-oxidising bacteria, and dominance of methane production via SAO (Westerholm et al. 2015). In the reactor operating with manure (reactor a), quantification of methanogens by qPCR illustrated dominance of order *Methanosarcinaceae* and *Methanobacteriales* and lower levels of *Methanomicrobiales*, suggesting a mix of methylotrophic and hydrogenotrophic methanogenesis. Analysis of the inoculum from the WWTP plant (reactor c) in a previous study showed it to be dominated by the acetoclastic genus *Methanosaeta,* a strict acetoclastic methanogen (Liu et al. 2017). The highest methane production from syngas was seen for inoculum A (from the manure-based reactor), which could have been caused by factors such as (i) the presence of both methylotrophic and hydrogenotrophic methanogenesis, since hydrogenotrophic methanogens mainly utilise hydrogen during syngas methanation but acetate can be produced via acetogenesis from CO, requiring acetoclastic methanogens for further conversion to methane and (ii) high abundance of order *Methanobacteriales*, as several studies on biomethanation in TBR have shown enrichment of methanogens within this order, such as genus *Methanobacterium* and *Methanothermobacter*, indicating importance for biomethanation in such reactors (Aryal et al. 2021; Sposob et al. 2021). Previous studies on syngas methanation in TBR have used inoculum from biogas processes operating with manure (Aryal et al. 2021) and sludge (Grimalt-Alemany et al. 2020; Figueras et al. 2021; Li et al. 2021), or a mix of both (Asimakopoulos et al. 2020a), as well as syngas- or H_2_-enriched cultures (Asimakopoulos et al. 2020b, 2021; Sieborg et al. 2020) and defined cultures comprising just a few organisms (Kimmel et al. 1991; Klasson et al. 1992). No obvious trends have emerged that some inocula are more suitable than others. Some thermophilic processes have even been initiated with mesophilic inocula but have still resulted in well-functioning processes (Kimmel et al. 1991; Grimalt-Alemany et al. 2020; Li et al. 2020b). However, for methanation of CO_2_ with H_2_, inoculation with enriched culture is reported to shorten the lag phase during start-up (Sposob et al. 2021). Microbiological studies of continuously operated TBR have observed a complete change in syngas-enriched communities compared with the inoculum and high adaptive capacity, likely due to intrinsic biological diversity (Asimakopoulos et al. 2020a; Grimalt-Alemany et al. 2020). Such a change was also observed in the present study and is discussed further below in the section ‘Microbial communities in the trickle bed reactor’.

### Methane productivity and VFA accumulation

Previous studies on productivity during biomethanation in TBR have reported different values, influenced by different parameters such as reactor design, carrier material, inflow gas composition and rate, nutrient composition and loading rate, gas injection, operating time and inoculum source (Asimakopoulos et al. 2020a; Grimalt-Alemany et al. 2020b; Aryal et al. 2021; Sposob et al. 2021). For methanation of gas composed of only H_2_ and CO_2_, values of around 1.17–3.1 L _CH4_/L/d during mesophilic (38–40 °C) operation have been reported (Burkhardt and Busch 2013; Burkhardt et al. 2015; Rachbauer et al. 2016). During thermophilic operations, considerably higher values, up to 8.85–15.4 L_CH4_/L/d, have been reported (Strübing et al. 2017; Lemmer and Ullrich 2018). For methanation from syngas, values between 0.21–1.90 and 1.88–9.46 L_CH4_/L/d for mesophilic (Grimalt-Alemany et al. 2018) and thermophilic conditions, respectively, have been reported. The higher productivity at higher temperatures is suggested to relate to higher conversion efficiencies resulting from increased methanogenic activity and abundance (Lemmer and Ullrich 2018; Asimakopoulos et al. 2020b). The TBR in the present study was operated under mesophilic conditions, and methane production was in line with that in previous studies operating at this temperature, reaching maximum values of 0.9–1.2 L_CH4_/L_pbv_/d. Production efficiency was lowest at the beginning of the process (period 1), mainly caused by difficulties in increasing the load of syngas due to accumulation of organic acids and low pH values. Accumulation of VFA indicates imbalances between the microbiological steps in digestion, with acid formation rate exceeding methanogenesis. Instances of acid accumulation have been observed previously during methanation of syngas, particularly in response to increasing levels of H_2_ in the syngas, and it is believed to be caused by inhibition of syntrophic acid conversion (Li et al. 2021). The acids produced in the present study were initially composed of both acetate and propionate but shifted towards a higher fraction of acetate at the end of the period. Acetate is the main product of acetogenesis, but none of the acetogenic carboxydotrophs isolated to date can produce propionate. However, in addition to acetate, acetogens can also produce ethanol and small amounts of butyrate, butanol and 2,3-butanediol, which in turn can be converted by other bacteria to propionate (Moreira et al. 2021). Organic acids were degraded in the second period of operation in this study (period 2), which allowed a higher syngas load and initially resulted in slightly increased methane productivity. Degradation of propionate proceeds via syntrophic collaboration and results in formation of acetate and hydrogen, which if present in high levels can block further degradation (Westerholm et al. 2021). Hydrogen is used by methanogens, but acetate can be converted via acetoclastic methanogens or via SAO (Westerholm et al. 2021; Sancho Navarro et al. 2016). No acetoclastic methanogens were detected in period 1, which might explain why acetate accumulated, although accumulation could also have been caused by decreased hydrogenotrophic methanogenic activity, causing problems for acetate degradation via SAO. Methanogenic activity can be inhibited by CO, VFA and low pH (Luo et al. 2013; Sancho Navarro et al. 2016; Grimalt-Alemany et al. 2018), which all appeared at the same time in period 1. The relative abundance of methanogens was lowest in periods 1B and 2A, which could have resulted in less efficient acid degradation. The improved VFA conversion observed in period 2 could have been caused by factors such as (i) acclimatisation of the methanogenic community to inhibiting conditions; (ii) installation of the anaerobic filter, prolonging the time for degradation and/or (iii) the change of nutrient medium, providing buffering capacity and additional nutrients for improved microbial growth or providing new microbes, including acetate-degrading microorganisms.

### Effect of nutrient source

Among the various parameters influencing the methanation processes and the activity of the microorganisms involved, the nutrient source is of crucial importance. For economic feasibility of full-scale applications, finding a cheap nutrient source is essential (Wegener Kofoed et al. 2021). Ideally, the nutrient medium should supply macronutrients and micronutrients, as well as buffering agents that can help to stabilise the pH in the event of acid formation (Sposob et al. 2021). For biological CO_2_ methanation, including TBR, several different non-defined cheap nutrient sources, such as digestate from different biogas processes and manure, have been evaluated and have been shown to be economically feasible for both mesophilic and thermophilic operation (for reviews, see Sposob et al. 2021; Wegener Kofoed et al. 2021). However, many previous studies have used defined nutrient medium for syngas methanation (Grimalt-Alemany et al. 2018; Asimakopoulos et al. 2020a, 2021; Grimalt-Alemany et al. 2020) and only a few have evaluated non-defined nutrients sources, mostly in batch systems (Ács et al. 2019; Aryal et al. 2021). To our knowledge, only one previous study has used a undefined nutrient source during continuous operation of a TBR for syngas methanation (Figueras et al. 2021). The batch reactors were operated under mesophilic conditions and with digestate from manure and wastewater processes and methane production reached only 0.15–0-22 L CH_4_/L/d. However, during continuous operation with dewatered WWTP digestate for more than 70 days under thermophilic conditions (55 °C), methane production values of 6.8 mmol CH_4_/L_reactor_/h, corresponding to 3.65 L L_CH4_/L_pbv_/d, have been reported (Figueras et al. 2021). This is three-fold the value obtained in the present study when using a similar nutrient source (in period 3 of operation). A likely explanation for the higher productivity is thus the higher temperature rather than the nutrient source, and possibly the higher pressure (~ 4 atm) applied in the previous study (Figueras et al. 2021) than in the present study (1.5 atm).

There is currently no consensus on the medium composition and origin that represent the best nutrient source for a biomethanation process (Wegener Kofoed et al. 2021). Nutrients suggested to be of specific importance for methanation from both H_2_/CO_2_ and syngas include macronutrients such as N, S and P and various trace elements, all of importance for methanogenic activity (Strübing et al. 2017; Li et al. 2020b; Figueras et al. 2021; Wegener Kofoed et al. 2021). According to the results in this and other studies, undefined nutrient sources of different origins can work well for biomethanation, offering the possibility to establish full-scale sustainable processes using cheap nutrient sources. A possible drawback/limitation with such nutrient sources is the need for pre-treatments to remove particles and to prevent growth of unintended microorganisms potentially also producing biogas from additional carbon sources (Sposob et al. 2021). In the present study, a shift was made from a defined nutrient medium to two different types of digestate. Neither of these digestates was sanitised before use, but the digestate from the food waste biogas plant (M2) had to be filtered before use. The process showed better performance during operation with the undefined nutrient sources than with the defined medium, with no acid accumulation and with the possibility for higher syngas loads. However, the defined medium was used in the initial phase of TBR operation and it cannot be concluded that this medium was less beneficial for the process, since process stabilisation and biofilm development may not have been complete. The periods with undefined medium occasionally suffered from low pH (with no VFA accumulation), particularly when the nutrient flow rate was lowered, leading to additional need for buffering capacity. Moreover, during most of the TBR operating time, the process was supported by additional S, in all phases, indicating a need for additional nutrients. During methanation of syngas in the study by Figueras et al. (2021), supplementation with additional S (Na_2_S) was found to be beneficial for the process. Similar findings were made in a previous study on biomethanation from H_2_/CO_2_ at thermophilic temperature using a TBR (Strübing et al. 2017). Strübing et al. (2017) suggested that sulphur deficiency could be caused by the loss of sulphide in the off-gas due to trickling of the liquid. These previously observed positive effects of sulphide addition were confirmed in the present study, where supplementation with additional sulphide was found to be beneficial for the conversion efficiency in periods 1A, 1B and 3A. Methanogens mainly use sulphide as a sulphur source, but some can also assimilate cysteine (Liu et al. 2012), and both were present in the defined medium (M1) in this study. In periods 2B and 3B, addition of sulphate instead was evaluated (Supplementary Material). Sulphate is not used directly by methanogens but can be converted to sulphide by sulphate-reducing bacteria present in the recycled nutrient medium. No obvious difference in methane productivity related to S source was however seen in this study.

### Microbial communities in the TBR

#### Methanogenic community

Throughout the process, independent of nutrient medium, one dominant methanogen was observed, represented by one ASV showing 100% similarity with *Methanobacterium bryantii*. Even though the digestate-based nutrient sources (M2 and M3) were not sterilised, methanogens from these sources did not establish in the process. Nutrient medium M2 was derived from a thermophilic biogas process, which might explain why no methanogens from this medium established in the mesophilic TBR. However, transition from thermophilic to mesophilic conditions with inoculum from the same biogas plant as M2 has been shown to be possible, illustrating the presence of mesophilic methanogenic species in this biogas plant (Westerholm et al. 2018). Nutrient medium M3 was derived from a mesophilic biogas plant, but both M2 and M3 showed little to no abundance of methanogens (representing less than 1% of the whole community), possibly also explaining the low contribution of methanogens to the methanation process in the TBR. The dominant methanogen, identified as *Methanobacterium bryantii*, is a hydrogenotrophic methanogen using H_2_/CO_2_, but not formate (Benstead et al. 1991). It is unclear whether this bacterium can use CO, but other members within this genus, e.g. the thermophilic *Methanobacterium thermoautotrophicus*, can grow with CO as the sole energy source, although at very low growth rates (Ferry 2010). Members within the genus *Methanobacterium* have been found to dominate in several other studies on biomethanation in TBR at both mesophilic and thermophilic temperatures (Rachbauer et al. 2017; Porté et al. 2019) and in other processes during ex situ and in situ biomethanation of H_2_ and syngas (Li et al. 2020a; Aryal et al. 2021; Jiang et al. 2021; Braga Nan et al. 2022). In line with results in the present study, this genus has also been found on carrier biofilm (Rachbauer et al. 2017; Thapa et al. 2021). Moreover, in several studies it has been observed under mesophilic conditions together with the hydrogenotrophic genus *Methanoculleus* and is suggested to be more crucial of the two in restoring efficiency after starvation periods and VFA accumulation, while also being decisive for efficient biomethanation due to high hydrogen consumption rates (Logroño et al. 2021; Braga Nan et al. 2022). *Methanoculleus*, a known partner during SAO and prevailing under low hydrogen concentrations (Westerholm et al. 2016), was not detected in the present study. In addition to *Methanobacterium*, genus *Methanosarcina* was observed in low relative abundance on the carrier biofilm in period 2B. In BLAST searches, this ASV was identified as *Methanosarcina flavescens*, a methanogen utilising both acetate and H_2_/CO_2_ for growth, and also methanol and methylamines (Kern et al. 2016). Establishment of this genus in period 2 could have occurred via the nutrient medium, as it has been identified previously at low abundance in the biogas plant from which the digestate originated (Westerholm et al. 2018). However, the genus could not be detected in the nutrient solution M2 (Fig. S3). In the process, *Methanosarcina* sp. could have used either acetate or H_2_/CO_2_, or both, but its enrichment in period 2B after the observed decrease in acetate concentration suggests that it acted as an acetoclastic methanogen. However, members of this genus, including *M. flavescens*, may be able to shift their metabolism from acetate to H_2_ in response to increasing partial pressure of H_2_, making them more competitive for hydrogen (Thapa et al. 2021). Thus, it is possible that *M. flavescens* acted as a hydrogenotrophic methanogen in the present study, together with *Methanobacterium*. Moreover, it is possible that *M. flavescens* used CO, as several species within genus *Methanosarcina* have been demonstrated to have this ability (Oelgeschläger and Rother 2008). In line with the results in this study, *M. flavescens* was recently identified at higher abundance in biofilm than in the liquid phase during in situ biogas upgrading in an anaerobic TBR treating thermal post-treated digestate (Thapa et al. 2021).

#### Bacterial community

The AVS identified as *Sporomusa sphaeroides* showed high abundance throughout the operation. This genus is known to utilise H_2_ and CO_2_, why it seems likely that this acetogen competed with the methanogen for its substrate. Some species within genus *Sporomusa* can also utilise CO, however, not *S. sphaeroides* (see review by Bengelsdorf et al. 2018). The end product of *Sporomusa* is mainly acetate, but *S. sphaeroides* can also produce small amount of ethanol (Möller et al. 1984). In addition to *Sporomusa*, one unknown member of *Spirochaetaceae* was highly abundant in the first phase of period 1. Members within *Spirochaetaceae* have been proposed to be involved in syntropic acid degradation, specifically acetate, together with methanogens (Wang et al. 2019). The abundance of this genus decreased/increased with observed acetate accumulation/consumption, indicating involvement in syntrophic acetate degradation also in this study. In the period with high VFA levels, increasing abundance of family *Rikenellaceae* and genera *Thermovirga* was also observed. Members within *Thermovirga* cannot use H_2_/CO_2_ or fatty acids but utilise proteinaceous substrate and amino acids, including cysteine, while producing acetate as the end product (Dahle and Birkeland 2006). This bacterium likely contributed to production of acetate, using yeast extract and/or the cysteine used as a reducing agent in nutrient medium M1. The abundance of this genus decreased to < 1% when the nutrient medium was changed in periods 2 and 3, but it was still found in biofilm on carrier samples from period 2, suggesting that also the digestates supported growth of this genus. Family *Rikenellaceae* contains several different genera, and species isolated so far can ferment carbohydrates or proteins and grow on yeast extract, while producing acetate and propionate and also H_2_ and CO_2_ and other acids (Krieg et al. 2010; Abe et al. 2012; Graf 2014; Su et al. 2014). Members of this family have been found previously in batch reactors fed with syngas (Aryal et al. 2021). In the present study, family *Rikenellaceae* was represented by two different genera, one of which (genus DMER64) is suggested to be a potential syntrophic propionate degrader (Lee et al. 2019). This is in line with the decreased abundance of this genus with decreasing propionate concentration. This genus possibly took over the role of propionate degrader from genus LNR_A2_18, within family *Cloacimonadaceae*, that was initially present in the TBR. This family is suggested to act also as a syntrophic propionate degrader and its disappearance has been shown to be accompanied by an increase in propionate (as in the present study) and to be an indicator of process disturbance (Klang et al. 2019; Singh et al. 2021)*.*

In period 2A, a drastic change in microbial community composition was seen. An immediate rise in several well-known thermophilic microorganisms was observed, i.e. genus *Defluviitoga* (phylum Thermotogae) and order MBA03 and DTU014 (phylum Firmicutes). This composition was very much influenced by the microbial composition of the nutrient medium, which changed in period 2 to thermophilic food waste digestate at a high flow rate. The observed organisms were highly abundant in the nutrient medium per se, and is also common in thermophilic biogas reactors (Westerholm et al. 2018; Dyksma et al. 2020). It has been suggested that members within these taxonomic groups perform carbohydrate fermentation and do not have the ability to use gaseous substrates, so most likely, they did not contribute to the methanation process. However, in contrast, genus W27, within family *Cloacimonadaceae*, was highly abundant in period 2A and not detected in the nutrient medium. As mentioned earlier, members within this taxonomic group are suggested to be involved in propionate degradation (Westerholm et al. 2021). However, it is difficult to predict the role of genus W27 in the present study, as it was highly abundant in period 2A after VFA had been degraded and disappeared in phase 2B. The high nutrient flow rate may have supplied the process with substrates for bacteria producing propionate, but kept to a low level by the genus W27. In period 2B, when the nutrient flow was reduced, there might not have been enough substrate to maintain the growth of this organism at a high level. In period 2B, no accumulation of acids was seen and the process appeared to be more stable than in period 1 (with defined nutrient medium). Degradation of propionate, and acetate, via syntrophic reactions, only proceeds at low P_H2_, and thus, the observed improved VFA degradation could potentially have been caused by a more efficient H_2_ turnover in period 2B as compared to 2A. However, looking at the hydrogen level in the gas out flow illustrated small differences in this regard between the periods. The more complex medium M2 may instead have allowed for more efficient acetate turnover by enrichment of the potential acetate oxidiser *Spirochaetaceae*. The improved acetate conversion might also be explained by the establishment of this organism on the carrier biofilm, which was observed in period 2B, but not 2A. However, in periods 1 and 2A, other members within phylum *Synergistetes*, also representing potential acetate oxidisers, were observed.

In period 3, when the nutrient solution was changed to digestate from a mesophilic wastewater biogas system, the microbial community initially maintained the same composition as at the end of period 2B, with dominance of *Methanobacterium*, *Sporomusa* and *Spirochaetaceae*. Approaching the end of period 3A, the *Spirochaetaceae* genus even became dominant in the community (55.5%). In addition, the potential propionate-degrading LNR_A2-18 (phylum *Cloacimonetes*) reappeared in the community. The high abundance of these two organisms likely contributed to the low acid level in this period of operation. However, on entering period 3B, *Spirochaetaceae* decreased in abundance (12.4–3.3%), possibly coinciding with a drop in pH since it is suggested to be favoured by slightly basic conditions (Lee et al. 2013). Moreover, in this period, an increased abundance of *Acetobacterium wieringae* was observed. This species can grow and produce acetate while consuming CO_2_ and H_2_ (Braun and Gottschalk 1982; Poehlein et al. 2016). A recently isolated novel strain of *Acetobacterium wieringae* is also able to grow on carbon monoxide (100% CO), producing mainly acetate as the end product (Arantes et al. 2020). Thus, in this phase of TBR operation, CO might have been used by this organism. The CO-utilising organisms in operating periods 1 and 2 were not identified, but the member within *Sporomusa* found in both periods could have been a CO utiliser.

In conclusion, methanation of syngas (56% H_2_, 30% CO, 14% CO_2_) in a TBR during long-term operation (862 days) was possible using different nutrient sources (defined nutrient medium, dewatered digestate from a thermophilic biogas plant treating food waste and reject water from a biogas plant at a wastewater treatment plant). The process reached maximum methane production levels of 0.9–1.15 L/L_PBV_/d, with some variation during operation, which corresponded to similar production levels as observed before at mesophilic conditions. The process showed some imbalance with accumulation of VFA in period 1, when the defined nutrient medium was used. However, concentrations declined in later operating periods with undefined medium and after introduction of an anaerobic filter to prolong nutrient recycling time.

For the microbial community, the overall trend within each period with different nutrient medium was stabilisation towards the same dominant species by the end of the period. Thus, the community was altered at the start of each period with the change in nutrient source, but after some time, it returned to the composition established prior to the change in nutrient medium. The main microbes observed included *Methanobacterium*, as the dominant methanogen, and the acetogen *Sporomusa*, as a dominant bacterial genus, both in liquid and on the carriers. These are both using hydrogen and carbon dioxide, while producing mainly methane and acetate, respectively, and have commonly been detected in various biomethanation processes before. Acetate was likely mainly converted via syntrophic acetate oxidation by an abundant representative within the genus *Spirochaetaceae* but could also have been directly converted to methane via *Methanosarcina*, present on the carriers. *Acetobacterium* also appeared later in the process and represent a potential CO-consuming acetogen.

## Supplementary Information

Below is the link to the electronic supplementary material.

Supplementary file1 (PDF 2618 KB)

Supplementary file2 (XLSX 711 KB)

## Data Availability

The sequence data generated and analysed from this study is made available in NCBI repository in BioProject PRJNA796200.

## References

[CR1] Abe K, Ueki A, Ohtaki Y, Kaku N, Watanabe K, Ueki K (2012). *Anaerocella delicata* gen. nov., sp. nov., a strictly anaerobic bacterium in the phylum *Bacteroidetes* isolated from a methanogenic reactor of cattle farms. J Gen Appl Microbiol.

[CR2] Ács N, Szuhaj M, Wirth R, Bagi Z, Maróti G, Rákhely G, Kovács KL (2019). Microbial community rearrangements in power-to-biomethane reactors employing mesophilic biogas digestate. Front Energy Res.

[CR3] Ahlberg-Eliasson K, Westerholm M, Isaksson S, Schnürer A (2021). Anaerobic digestion of animal manure and influence of organic loading rate and temperature on process performance, microbiology, and methane emission from digestates. Front Energy Res.

[CR4] Andreides D, Bautista Quispe JI, Bartackova J, Pokorna D, Zabranska J (2021). A novel two-stage process for biological conversion of syngas to biomethane. Bioresour Technol.

[CR5] Arantes AL, Moreira JPC, Diender M, Parshina SN, Stams AJM, Alves MM, Alves JI, Sousa DZ (2020). Enrichment of anaerobic syngas-converting communities and isolation of a novel carboxydotrophic *Acetobacterium wieringae* strain JM. Front Microbiol.

[CR6] Aryal N, Odde M, Petersen CB, Ottosen LDM, Kofoed MVW (2021). Methane production from syngas using a trickle-bed reactor setup. Bioresour Technol.

[CR7] Asimakopoulos K, Gavala HN, Skiadas IV (2020). Biomethanation of syngas by enriched mixed anaerobic consortia in trickle bed reactors. Waste Biomass Valorization.

[CR8] Asimakopoulos K, Łężyk M, Grimalt-Alemany A, Melas A, Wen Z, Gavala HN, Skiadas IV (2020b) Temperature effects on syngas biomethanation performed in a trickle bed reactor. Chem Eng J 393.10.1016/j.cej.2020.124739

[CR9] Asimakopoulos K, Kaufmann-Elfang M, Lundholm-Høffner C, Rasmussen NBK, Grimalt-Alemany A, Gavala HN, Skiadas IV (2021) Scale up study of a thermophilic trickle bed reactor performing syngas biomethanation. Appl Energy 290.10.1016/j.apenergy.2021.116771

[CR10] Bengelsdorf FR, Beck MH, Erz C, Hoffmeister S, Karl MM, Riegler P, Wirth S, Poehlein A, Weuster-Botz D, Durre P (2018). Bacterial anaerobic synthesis gas (syngas) and CO2+H2 fermentation. Adv Appl Microbiol.

[CR11] Benjaminsson G, Benjaminsson J, Rudberg RB (2013) Power to gas – a technical review. SGC Rapport (2013:284). http://www.sgc.se/ckfinder/userfiles/files/SGC284_eng.pdf

[CR12] Benstead J, Archer DB, Lloyd D (1991). Formate utilization by members of the genus *Methanobacterium*. Arch Microbiol.

[CR13] Braga Nan L, Trably E, Santa-Catalina G, Bernet N, Delgenes J-P, Escudie R (2022). Microbial community redundance in biomethanation systems lead to faster recovery of methane production rates after starvation. Sci Total Environ.

[CR14] Braun M, Gottschalk G (1982). *Acetobacterium wieringae* sp. nov., a new species producing acetic acid from molecular hydrogen and carbon dioxide. Zbl Bakt Mik Hyg I C.

[CR15] Burkhardt M, Busch G (2013). Methanation of hydrogen and carbon dioxide. Appl Energy.

[CR16] Burkhardt M, Koschack T, Busch G (2015). Biocatalytic methanation of hydrogen and carbon dioxide in an anaerobic three-phase system. Bioresour Technol.

[CR17] Ciliberti C, Biundo A, Albergo R, Agrimi G, Braccio G, de Bari I, Pisano I (2020) Syngas derived from lignocellulosic biomass gasification as an alternative resource for innovative bioprocesses. Processes 8(12). 10.3390/pr8121567

[CR18] Dahle H, Birkeland N-K (2006). *Thermovirga lienii* gen. nov., sp. nov., a novel moderately thermophilic, anaerobic, amino-acid-degrading bacterium isolated from a North Sea oil well. Int J Syst Evol Microbiol.

[CR19] Danielsson R, Dicksved J, Sun L, Gonda H, Müller B, Schnürer A, Bertilsson J (2017). Methane production in dairy cows correlates with rumen methanogenic and bacterial community structure. Front Microbiol.

[CR20] De Vrieze J, Saunders AM, He Y, Fang J, Nielsen PH, Verstraete W, Boon N (2015). Ammonia and temperature determine potential clustering in the anaerobic digestion microbiome. Water Res.

[CR21] Dyksma S, Jansen L, Gallert C (2020). Syntrophic acetate oxidation replaces acetoclastic methanogenesis during thermophilic digestion of biowaste. Microbiome.

[CR22] Ferry JG (2010). CO in methanogenesis. Ann Microbiol.

[CR23] Figueras J, Benbelkacem H, Dumas C, Buffiere P (2021). Biomethanation of syngas by enriched mixed anaerobic consortium in pressurized agitated column. Bioresour Technol.

[CR24] Fu B, Jin X, Conrad R, Liu H, Liu H (2019). Competition between chemolithotrophic acetogenesis and hydrogenotrophic methanogenesis for exogenous H2/CO2 in anaerobically digested sludge: impact of temperature. Front Microbiol.

[CR25] Graf J (2014) The family *Rikenellaceae*. ( The Prokaryotes. ). Springer, Berlin, Heidelberg. 10.1007/978-3-642-38954-2_134

[CR26] Grim J, Malmros P, Schnürer A, Nordberg Å (2015). Comparison of pasteurization and integrated thermophilic sanitation at a full-scale biogas plant – heat demand and biogas production. Energy.

[CR27] Grimalt-Alemany A, Asimakopoulos K, Skiadas IV, Gavala HN (2020). Modeling of syngas biomethanation and catabolic route control in mesophilic and thermophilic mixed microbial consortia. Appl Energy.

[CR28] Grimalt-Alemany A, Łężyk M, Kennes-Veiga DM, Skiadas IV, Gavala HN (2020). Enrichment of mesophilic and thermophilic mixed microbial consortia for syngas biomethanation: the role of kinetic and thermodynamic competition. Waste Biomass Valorization.

[CR29] Grimalt-Alemany A, Skiadas IV, Gavala HN (2018). Syngas biomethanation: state-of-the-art review and perspectives. Biofuel Bioprod Bioresour.

[CR30] Grimalt-Alemany A, Skiadas IV, Gavala HN (2017). Syngas biomethanation: state-of-the-art review and perspectives. Biofuel Bioprod Bioresour.

[CR31] Hendriks AT, Zeeman G (2009). Pretreatments to enhance the digestibility of lignocellulosic biomass. Bioresour Technol.

[CR32] Jarrell KF, Kalmokoff ML (1988). Nutritional requirements of the methanogenic *archaebacteria*. Can J Microbiol.

[CR33] Jiang H, Wu F, Wang Y, Feng L, Zhou H, Li Y (2021). Characteristics of in-situ hydrogen biomethanation at mesophilic and thermophilic temperatures. Bioresour Technol.

[CR34] Kern T, Fischer MA, Deppenmeier U, Schmitz RA, Rother M (2016). *Methanosarcina flavescens* sp. nov., a methanogenic archaeon isolated from a full-scale anaerobic digester. Int J Syst Evol Microbiol.

[CR35] Kimmel DE, Klasson KT, Clausen EC, Gaddy JL (1991). Performance of trickle-bed bioreactors for converting synthesis gas to methane. Appl Biochem Biotechnol.

[CR36] Klang J, Szewzyk U, Bock D, Theuerl S (2019). Nexus between the microbial diversity level and the stress tolerance within the biogas process. Anaerobe.

[CR37] Klasson KT, Ackerson MD, Clausen EC, Gaddy JL (1992). Bioconversion of synthesis gas into liquid or gaseous fuels. Enzyme Microb Technol.

[CR38] Kougias PG, Angelidaki I (2018). Biogas and its opportunities—a review. Front Environ Sci Eng.

[CR39] Krieg NR, Ludwig W, Euzéby J, Whitman WB, Krieg NR, Staley JT, Brown DR, Hedlund BP, Paster BJ, Ward NL, Ludwig W, Whitman WB (2010). Phylum XIV. Bacteroidetes phyl. nov. Bergey’s Manual® of Systematic Bacteriology: Volume Four The Bacteroidetes, Spirochaetes, Tenericutes (Mollicutes), Acidobacteria, Fibrobacteres, Fusobacteria, Dictyoglomi, Gemmatimonadetes, Lentisphaerae, Verrucomicrobia, Chlamydiae, and Planctomycetes.

[CR40] Lee J, Koo T, Yulisa A, Hwang S (2019). Magnetite as an enhancer in methanogenic degradation of volatile fatty acids under ammonia-stressed condition. J Environ Manage.

[CR41] Lee S-H, Park J-H, Kang H-J, Lee Y, Lee T, Park H-D (2013) Distribution and abundance of Spirochaetes in full-scale anaerobic digesters. Bioresour Technol 145.10.1016/j.biortech.2013.02.070 10.1016/j.biortech.2013.02.070 23562175

[CR42] Lemmer A, Ullrich T (2018). Effect of different operating temperatures on the biological hydrogen methanation in trickle bed reactors. Energies.

[CR43] Li C, Zhu X, Angelidaki I (2020). Carbon monoxide conversion and syngas biomethanation mediated by different microbial consortia. Bioresour Technol.

[CR44] Li Y, Wang Z, He Z, Luo S, Su D, Jiang H, Zhou H, Xu Q (2020). Effects of temperature, hydrogen/carbon monoxide ratio and trace element addition on methane production performance from syngas biomethanation. Bioresour Technol.

[CR45] Li C, Zhu X, Angelidaki I (2021). Syngas biomethanation: effect of biomass-gas ratio, syngas composition and pH buffer. Bioresour Technol.

[CR46] Liu R, Hao X, Wei J (2016). Function of homoacetogenesis on the heterotrophic methane production with exogenous H2/CO2 involved. Chem Eng J.

[CR47] Liu T, Sun L, Müller B, Schnürer A (2017). Importance of inoculum source and initial community structure for biogas production from agricultural substrates. Bioresour Technol.

[CR48] Liu Y, Beer LL, Whitman WB (2012). Methanogens: a window into ancient sulfur metabolism. Trends Microbiol.

[CR49] Logroño W, Popp D, Nikolausz M, Kluge P, Harms H, Kleinsteuber S (2021). Microbial communities in flexible biomethanation of hydrogen are functionally resilient upon starvation. Front Microbiol.

[CR50] Luo G, Wang W, Angelidaki I (2013). Anaerobic digestion for simultaneous sewage sludge treatment and co biomethanation: process performance and microbial ecology. Environ Sci Technol.

[CR51] McMurdie PJ, Holmes S (2013). phyloseq: an R package for reproducible interactive analysis and graphics of microbiome census data. PLoS ONE.

[CR52] Möller B, Oßmer R, Howard BH, Gottschalk G, Hippe H (1984). *Sporomusa*, a new genus of gram-negative anaerobic bacteria including *Sporomusa sphaeroides* spec. nov. and *Sporomusa ovata* spec. nov. Arch Microbiol.

[CR53] Moreira JPC, Diender M, Arantes AL, Boeren S, Stams AJM, Alves MM, Alves JI, Sousa DZ (2021). Propionate production from carbon monoxide by synthetic cocultures of *Acetobacterium wieringae* and Propionigenic Bacteria. Appl Environ Microbiol.

[CR54] Oelgeschläger E, Rother M (2008). Carbon monoxide-dependent energy metabolism in anaerobic bacteria and archaea. Arch Microbiol.

[CR55] Paul A, Dutta A (2018). Challenges and opportunities of lignocellulosic biomass for anaerobic digestion. Resour Conserv Recyl.

[CR56] Poehlein A, Bengelsdorf FR, Schiel-Bengelsdorf B, Daniel R, Dürre P (2016). Genome sequence of the acetogenic bacterium *Acetobacterium wieringae DSM 1911T*. Genome Announc.

[CR57] Porté H, Kougias PG, Alfaro N, Treu L, Campanaro S, Angelidaki I (2019). Process performance and microbial community structure in thermophilic trickling biofilter reactors for biogas upgrading. Sci Total Environ.

[CR58] Rachbauer L, Beyer R, Bochmann G, Fuchs W (2017). Characteristics of adapted hydrogenotrophic community during biomethanation. Sci Total Environ.

[CR59] Rachbauer L, Voitl G, Bochmann G, Fuchs W (2016). Biological biogas upgrading capacity of a hydrogenotrophic community in a trickle-bed reactor. Appl Energy.

[CR60] Ren J, Liu Y-L, Zhao X-Y, Cao J-P (2020). Methanation of syngas from biomass gasification: an overview. Int J Hydrogen Energy.

[CR61] RStudio Team (2020) RStudio: Integrated Development for R. RStudio, PBC, Boston, MA. http://www.rstudio.com/

[CR62] Sancho Navarro S, Cimpoia R, Bruant G, Guiot SR (2016). Biomethanation of Syngas using anaerobic sludge: shift in the catabolic routes with the CO partial pressure increase. Front Microbiol.

[CR63] Sieborg MU, Jønson BD, Ashraf MT, Yde L, Triolo JM (2020). Biomethanation in a thermophilic biotrickling filter using cattle manure as nutrient media. Bioresour Technol Rep.

[CR64] Singh A, Müller B, Schnürer A (2021). Profiling temporal dynamics of acetogenic communities in anaerobic digesters using next-generation sequencing and T-RFLP. Sci Rep.

[CR65] Sposob M, Wahid R, Fischer K (2021). Ex-situ biological CO2 methanation using trickle bed reactor: review and recent advances. Rev Environ Sci Biotechnol.

[CR66] Strübing D, Huber B, Lebuhn M, Drewes JE, Koch K (2017). High performance biological methanation in a thermophilic anaerobic trickle bed reactor. Bioresour Technol.

[CR67] Su X-L, Tian Q, Zhang J, Yuan X-Z, Shi X-S, Guo R-B, Qiu Y-L (2014). *Acetobacteroides hydrogenigenes gen.* nov., sp. nov., an anaerobic hydrogen-producing bacterium in the family *Rikenellaceae* isolated from a reed swamp. Int J Syst Evol Microbiol.

[CR68] Sundberg C, Al-Soud WA, Larsson M, Alm E, Yekta SS, Svensson BH, Sørensen SJ, Karlsson A (2013). 454 pyrosequencing analyses of bacterial and archaeal richness in 21 full-scale biogas digesters. FEMS Microb Ecol.

[CR69] Thapa A, Park J-G, Yang H-M, Jun H-B (2021). In-situ biogas upgrading in an anaerobic trickling filter bed reactor treating a thermal post-treated digestate. J Environ Chem Eng.

[CR70] Tsapekos P, Treu L, Campanaro S, Centurion VB, Zhu X, Peprah M, Zhang Z, Kougias PG, Angelidaki I (2021) Pilot-scale biomethanation in a trickle bed reactor: process performance and microbiome functional reconstruction. Energy Convers Manage 244.10.1016/j.enconman.2021.114491

[CR71] Voelklein MA, Rusmanis D, Murphy JD (2019). Biological methanation: Strategies for in-situ and ex-situ upgrading in anaerobic digestion. Appl Energy.

[CR72] Wang H-Z, Lv X-M, Yi Y, Zheng D, Gou M, Nie Y, Hu B, Nobu MK, Narihiro T, Tang Y-Q (2019). Using DNA-based stable isotope probing to reveal novel propionate- and acetate-oxidizing bacteria in propionate-fed mesophilic anaerobic chemostats. Sci Rep.

[CR73] Wegener Kofoed MV, Jensen MB, Mørck Ottosen LD (2021) Chapter 12 - Biological upgrading of biogas through CO2 conversion to CH4. In: Aryal N, Mørck Ottosen LD, Wegener Kofoed MV, Pant D (eds) Emerging technologies and biological systems for biogas upgrading. Academic Press; pp 321–362. [2022–01–12 08:38:14]

[CR74] Westerholm M, Calusinska M, Dolfing J (2021) Syntrophic propionate-oxidizing bacteria in methanogenic systems. FEMS Microbiol Rev:fuab057. 10.1093/femsre/fuab057 10.1093/femsre/fuab057 PMC8892533 34875063

[CR75] Westerholm M, Hansson M, Schnürer A (2012). Improved biogas production from whole stillage by co-digestion with cattle manure. Bioresour Technol.

[CR76] Westerholm M, Isaksson S, Karlsson Lindsjö O, Schnürer A (2018). Microbial community adaptability to altered temperature conditions determines the potential for process optimisation in biogas production. Appl Energy.

[CR77] Westerholm M, Moestedt J, Schnürer A (2016). Biogas production through syntrophic acetate oxidation and deliberate operating strategies for improved digester performance. Appl Energy.

[CR78] Westerholm M, Müller B, Isaksson S, Schnürer A (2015). Trace element and temperature effects on microbial communities and links to biogas digester performance at high ammonia levels. Biotechnol Biofuels.

[CR79] Westerholm M, Roos S, Schnürer A (2010). *Syntrophaceticus schinkii* gen. nov., sp. nov., an anaerobic, syntrophic acetate-oxidizing bacterium isolated from a mesophilic anaerobic filter. FEMS Microbiol Lett.

