# Supplementary material for: Microbial community development during syngas methanation in a trickle bed reactor with various nutrient sources
Source: Appl Microbiol Biotechnol. 2022 Jul 8;106(13-16):5317–33. doi: 10.1007/s00253-022-12035-5 (PMC9329420; doi:10.1007/s00253-022-12035-5)
Supplement: Supplementary file 1 — Supplementary file1 (PDF 2618 KB) [file 253_2022_12035_MOESM1_ESM.pdf]

Applied Microbiology and Biotechnology

Supplementary Material

for

Microbial community development during syngas methanation in a trickle bed reactor with various nutrient sources

George Cheng[1], Florian Gabler[2,3], Leticia Pizzul[3], Henrik Olsson[3], Åke Nordberg[2,3], and Anna Schnürer[1*]

[1]Department of Molecular Science, Biocenter SLU, Box 7015, 750 07 Uppsala, Sweden

[2]Department of Energy and Technology, SLU, Box 7032, 750 07 Uppsala, Sweden

[3]Department of Biorefinery and Energy, RISE, Box 7033, 750 07 Uppsala, Sweden

Correspondence: Anna Schnürer, email: anna.schnurer@slu.se; Tel: +4618673288

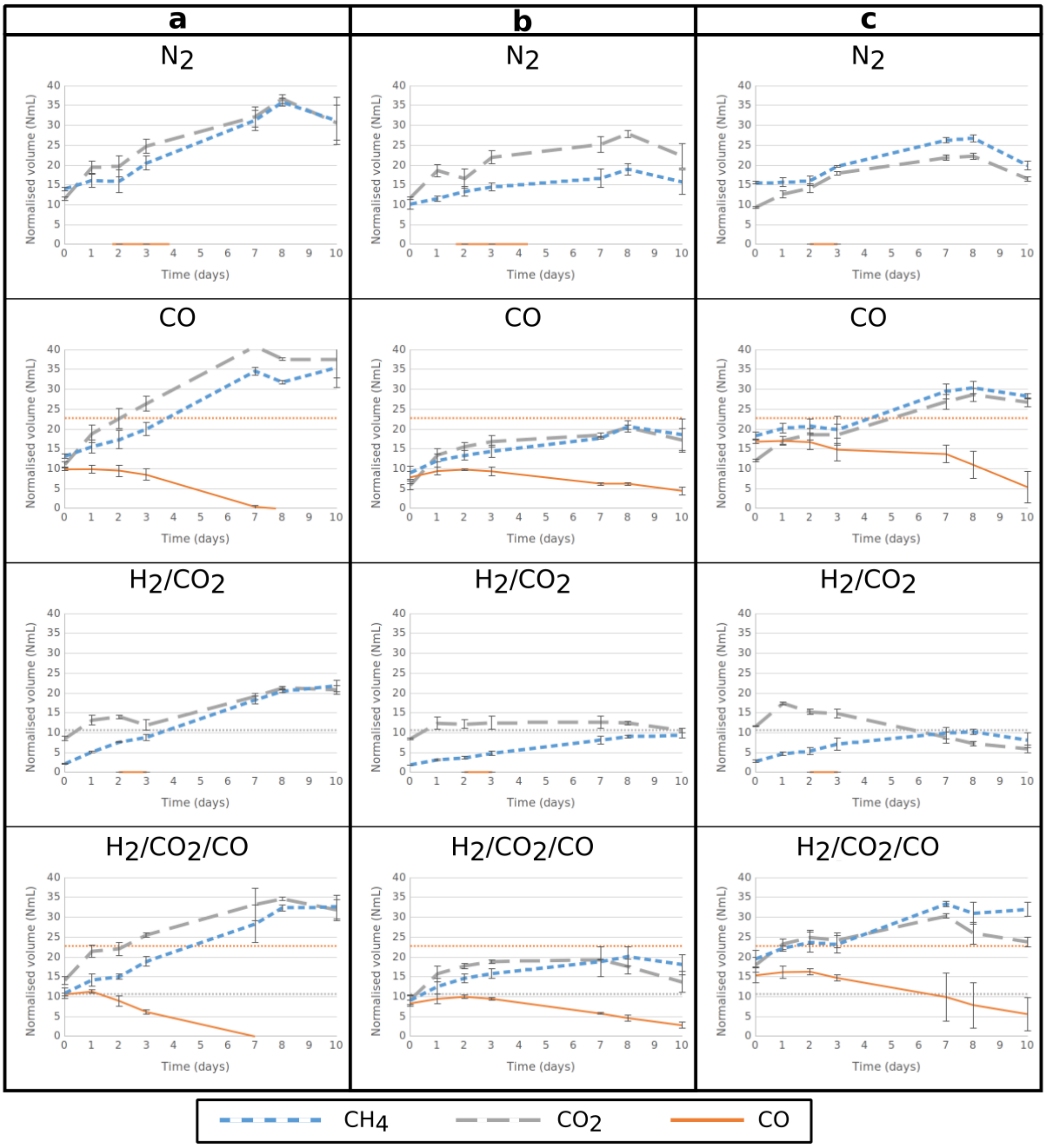

Fig. S1 Performance of reactors in initial screening of different digestates for use as inoculum: **a)** cow manure, **b)** mixed food waste and **c)** digestate from a wastewater treatment plant. The reactor bottles were capped and filled with different gases to the following gas composition at 1.5 atm: N_2 (100%), CO/N_2 (15/85%), H_2/CO_2 (28/72%), $H_2/CO_2/CO/N_2$ (28/7/15/50%)

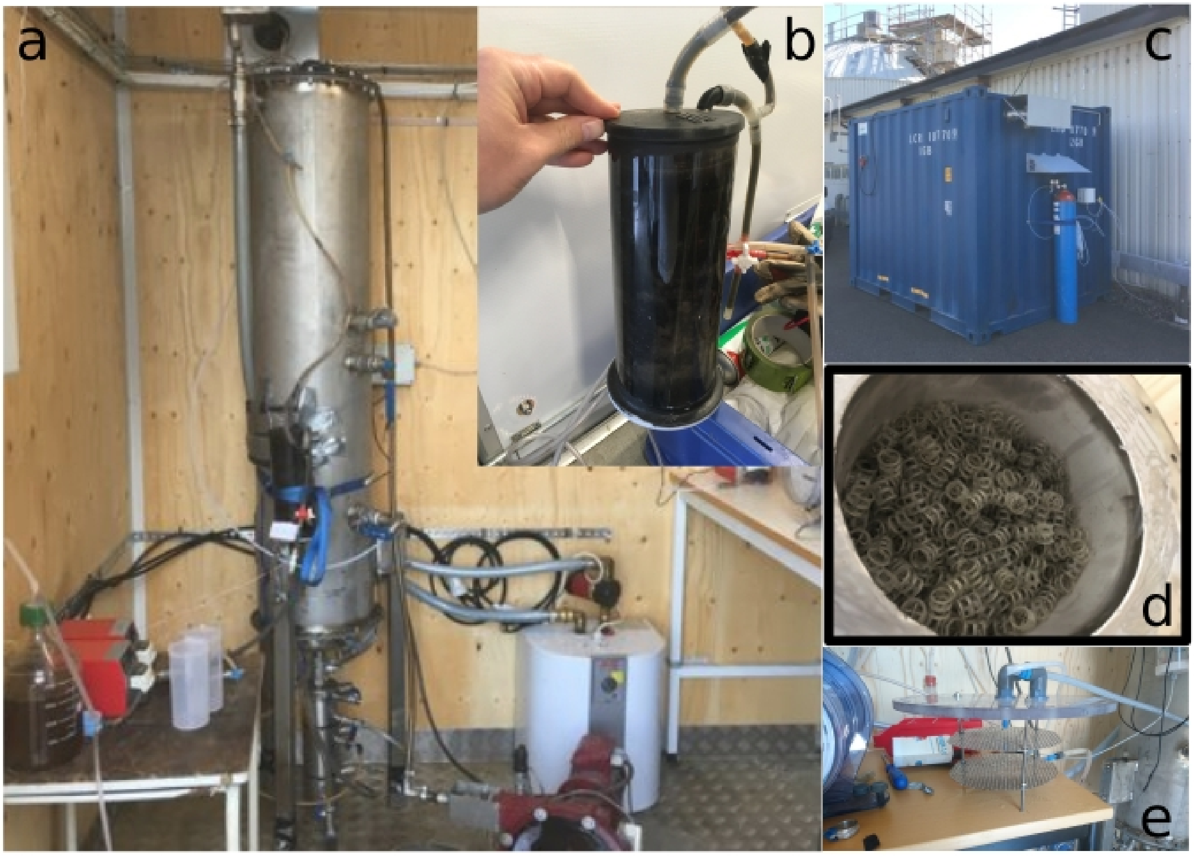

Fig. S2 a) Trickle bed reactor (TBR) (gray/silver column on the left) with a water heater (white) near the foot of the TBR to maintain internal temperature. **b)** Anaerobic filter reactor. **c)** Shipping container that housed the TBR system. **d)** Plastic carrier within the TBR. **e)** Spreader plate to evenly disperse nutrient solution to the TBR

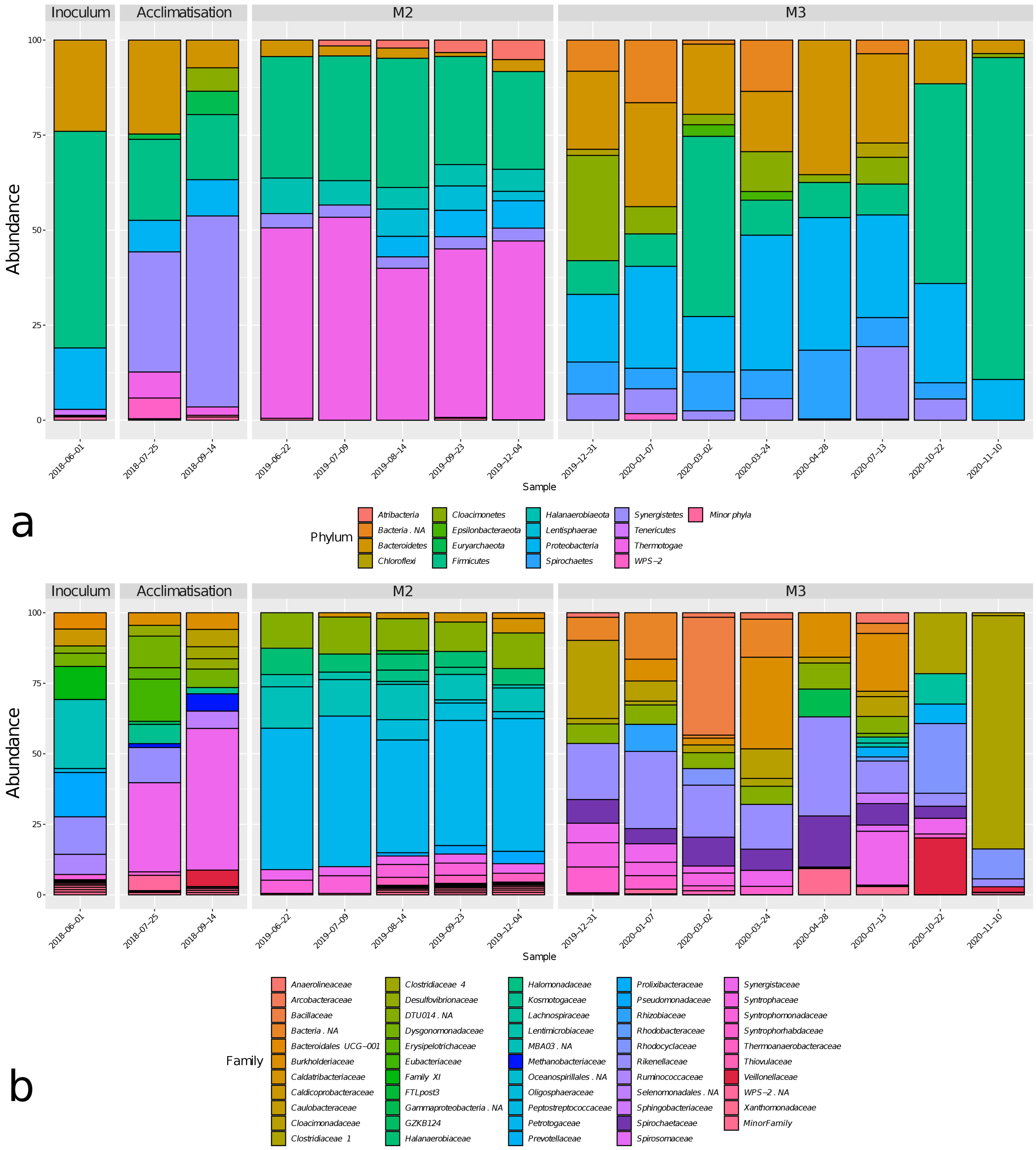

Fig. S3 Microbial community structure at **a)** phylum level and **b)** family level in the inoculum and in nutrient solution added during operation of the trickle bed reactor (TBR). Acclimatisation samples were taken from nutrient solution recycled in the TBR. M2 (period 2): dewatered digestate from a thermophilic biogas plant operating with food waste M3 (period 3): reject water from a biogas plant at a wastewater treatment plant

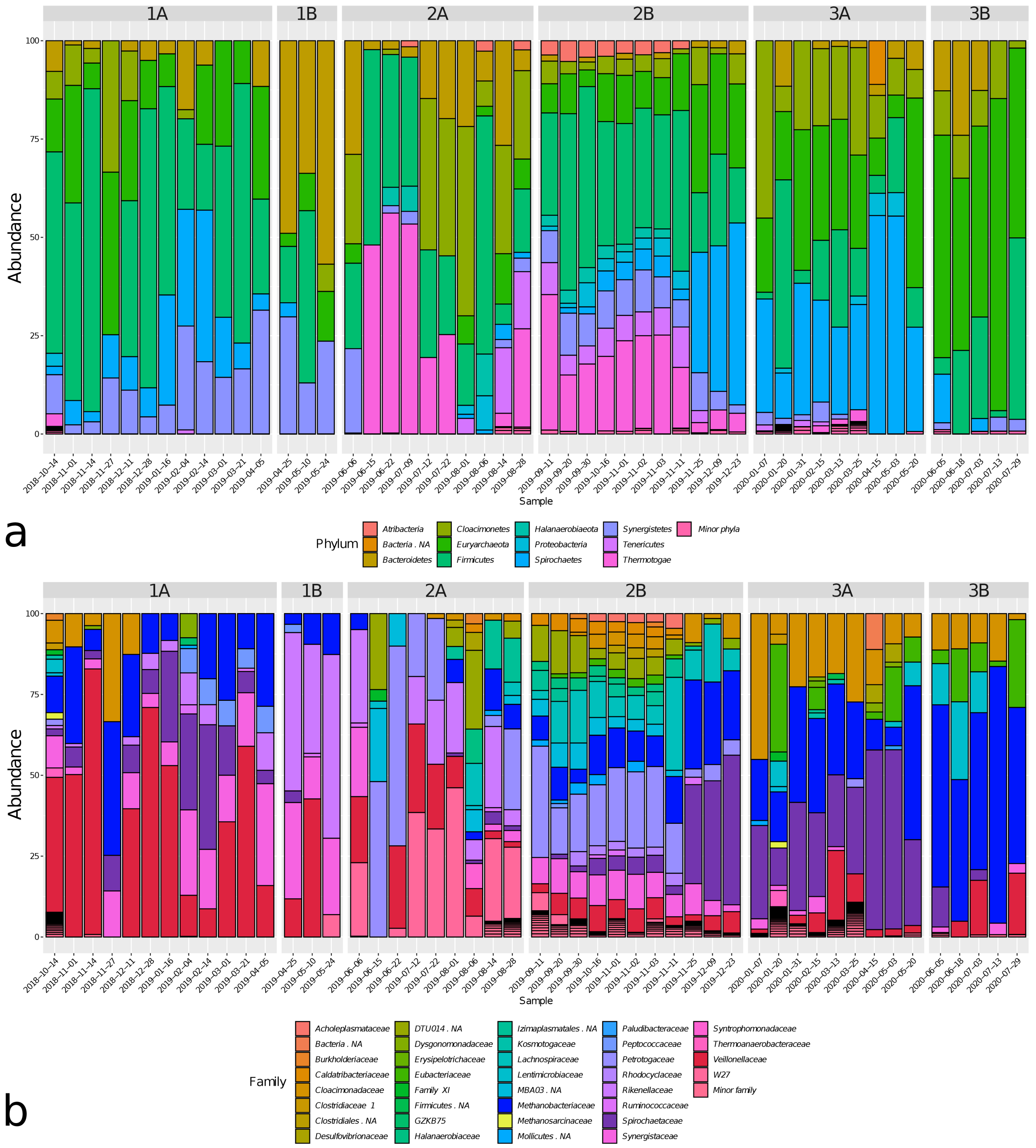

Fig. S4 Microbial community structure at **a**) phylum level and **b**) family level during operation of a trickle bed reactor (TBR) in three periods (1-3) with different nutrient medium: 1) defined mineral medium (M1), 2) dewatered digestate from a thermophilic biogas plant operating with food waste (M2) and 3) reject water from a biogas plant at a wastewater treatment plant (M3). Each operating period was further divided into two sub-phases (A, B) based on major changes in operating parameters, such as flow rate of nutrient medium (see Table 1)

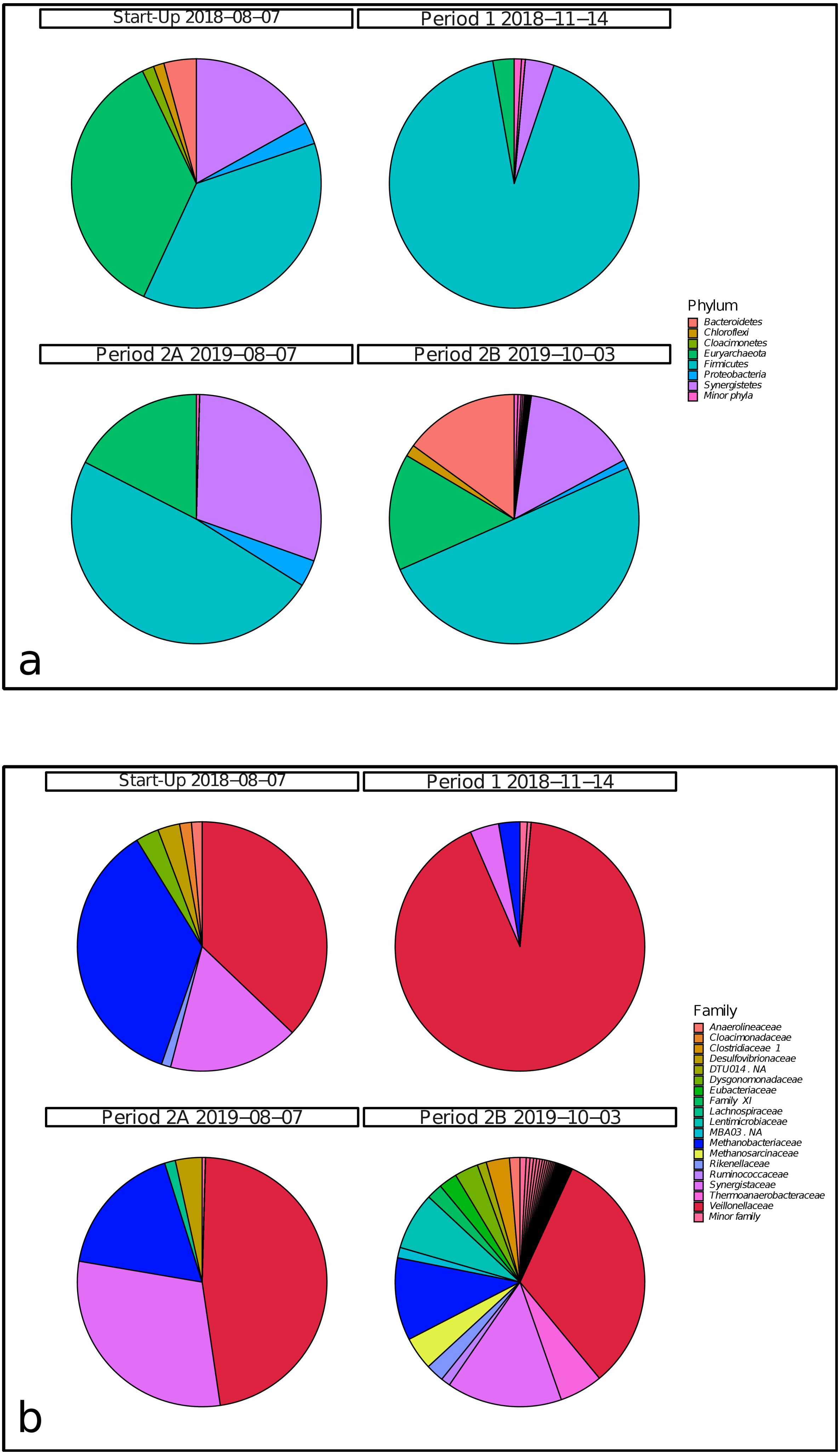

Start-Up 2018–08–07
Period 1 2018–11–14
Period 2A 2019–08–07
Period 2B 2019–10–03
Phylum
Bacteroidetes
Chloroflexi
Cloacimonetes
Euryarchaeota
Firmicutes
Proteobacteria
Synergistetes
Minor phyla
a
Start-Up 2018–08–07
Period 1 2018–11–14
Period 2A 2019–08–07
Period 2B 2019–10–03
Family
Anaerolineaceae
Cloacimonadaceae
Clostridiaceae 1
Desulfovibrionaceae
DTU014 . NA
Dysgonomonadaceae
Eubacteriaceae
Family XI
Lachnospiraceae
Lentimicrobiaceae
MBA03 . NA
Methanobacteriaceae
Methanosarcinaceae
Rikenellaceae
Ruminococcaceae
Synergistaceae
Thermoanaerobacteraceae
Veillonellaceae
Minor family
b

Fig. S5 Microbial community at **a)** phylum level and **b)** family level on samples of plastic carrier taken from the trickle bed reactor (TBR) reactor in the start-up period and in operating periods 1 and 2. Carrier samples taken in Period 2B were sequenced and processed in triplicate. Carrier samples taken in start-up, period 1 and period 2A were sequenced without replicates, due to lack of extracted material.
